# Infection prophylaxis following anti-CD20 monoclonal antibodies in childhood kidney diseases

**DOI:** 10.1007/s00467-026-07180-2

**Published:** 2026-02-23

**Authors:** Dongyang Zhou, Fiona Fung-Yee Lai, Joshua Sung-Chih Wong, Eugene Yu-Hin Chan, Alison Lap-Tak Ma

**Affiliations:** 1 Department of Paediatrics, Faculty of Medicine, The Chinese University of Hong Kong, Shatin, Hong Kong SAR; 2 Department of Pharmacy, Hong Kong Children’s Hospital, Kowloon Bay, Hong Kong SAR; 3 Department of Paediatrics and Adolescent Medicine, Princess Margaret Hospital, Kowloon, Hong Kong SAR; 4 Paediatric Nephrology Centre, Hong Kong Children’s Hospital, Kowloon Bay, Hong Kong SAR

**Keywords:** Anti-CD20, Kidney diseases, Children, Infection, Rituximab, Obinutuzumab

## Abstract

Anti-CD20 monoclonal antibodies (mAbs), which act as B-cell–depleting therapies, are now regarded as important treatments for a range of paediatric kidney diseases. Despite their effectiveness in achieving B-cell depletion and consequent disease remission, concerns remain regarding their side effect profile. These include infusion reactions, hypogammaglobulinaemia, and neutropenia, as well as infections. At present, the evidence supporting preventive measures such as intravenous immunoglobulin (IVIG) replacement and antibiotic prophylaxis remains inconclusive. In this review, we summarise the existing data on infection-related risks following anti-CD20 therapy and propose practical strategies to mitigate infection risk tailored to this group of children.

**Graphical Abstract:**

**Figure:**
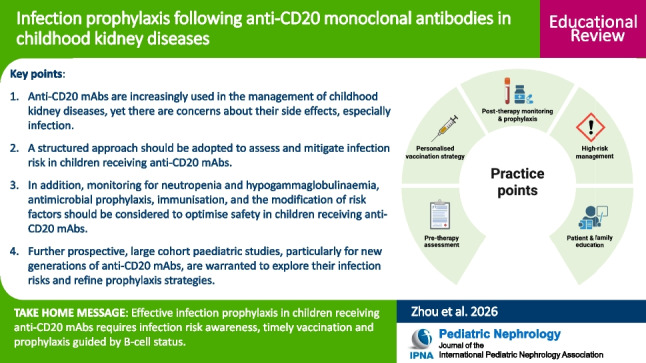
A higher resolution version of the Graphical abstract is available as Supplementary information

**Supplementary information:**

The online version contains supplementary material available at 10.1007/s00467-026-07180-2.

## Introduction

Anti-CD20 monoclonal antibodies (mAbs) are a B-cell depleting therapy that depletes the B-cell population [1]. Their clinical use was first described in B-cell non-Hodgkin’s lymphoma [2]. Subsequently, they have been widely utilised in various autoimmune conditions, including rheumatoid arthritis and ANCA-associated vasculitis (AAV) [3, 4]. In paediatric nephrology, one of the main indications for anti-CD20 mAbs is steroid-sensitive nephrotic syndrome (SSNS). Multiple clinical trials established the efficacy of anti-CD20 mAbs in maintaining disease remission [5–8], and since then, they have become one of the important steroid-sparing approaches in steroid-dependent nephrotic syndrome (SDNS) [9–12]. Anti-CD20 mAbs have been increasingly utilised for other childhood kidney diseases such as steroid-resistant nephrotic syndrome, lupus nephritis (LN), AAV, antibody-mediated rejection (AMR) of graft kidney, and membranous nephropathy (MN) [13–18] (Supplementary Table 1).

Although anti-CD20 mAbs, in particular rituximab, are generally considered to be safe in children, there are valid concerns about side effects. These range from infusion reactions, infection, hypogammaglobulinaemia, neutropenia, to severe and fatal complications related to hepatitis reactivation, myocarditis and multifocal leukoencephalopathy [19–23]. Infection is a significant concern, as approximately half of the children treated with rituximab develop hypogammaglobulinaemia [24], and concurrent immunosuppression and agranulocytosis post-rituximab can also contribute to infectious complications [25]. Data pertaining to preventive measures such as intravenous immunoglobulin (IVIG) supplementation and antibiotic prophylaxis remain scarce [26]. By comprehending the mechanisms of action of anti-CD20 treatment and the infections linked to their actions, clinicians can more effectively anticipate these risks and proactively prevent and identify opportunistic infections. In this review, we summarise the current understanding on infection-related risks associated with anti-CD20 mAbs in the management of paediatric kidney diseases, and propose practical preventive strategies to mitigate the risk in this specific patient population.

## Mechanism of action

Anti-CD20 mAbs target CD20 antigens expressed on B cells, mostly pre-B cells, transitional, naïve and memory B cells [1]. They are classified as type I (e.g. rituximab and ofatumumab, the latter no longer commercially available) and type II (e.g. obinutuzumab) [27]. Rituximab mediates B cell destruction through different mechanisms, including complement-dependent cytotoxicity, antibody-dependent cellular cytotoxicity, and direct cell death [2]. The dosing of rituximab ranged from 375 to 1500mg/m^2^ per treatment course, administered as 1 to 4 infusions, depending on the underlying conditions [9–11, 13–16]. The measurement of rituximab drug level does not predict treatment response or correlate with treatment toxicity [28]. At present, this medication remains off-label for paediatric use, and the optimal dosing regimen has yet to be defined by future trials. Obinutuzumab, a glycoengineered, second-generation anti-CD20 mAb, has been designed to overcome potential rituximab resistance by exhibiting a higher affinity for CD20 and enhanced B-cell depletion capacity [29]. The differences between rituximab and obinutuzumab are summarised in Table 1. Data pertaining to obinutuzumab in treating childhood kidney diseases are scarce; only a small number of retrospective data have been published among children with SDNS who were refractory or intolerant to rituximab [29, 31].

**Table 1:** Characteristics of Type I and II anti-CD20 monoclonal antibodies

	Type I	Type II
Mechanism of action	Induce the aggregation of CD20 into lipid rafts	Do not induce the aggregation of CD20 into lipid rafts
	High CDC	Low CDC
	ADCC	ADCC
	ADCP	ADCP
	Direct cell death	Stronger direct cell death
	B cell depletion	B cell depletion with longer duration [29]
FDA approved for paediatric kidney disease	AAV (≥ 2 years old) [30]	——
Examples	Rituximab, ofatumumab	Obinutuzumab
Availability as biosimilars	Available for rituximab	No

*CDC* complement-dependent cytotoxicity, *ADCC* antibody-dependent cellular cytotoxicity, *ADCP* antibody-dependent cellular phagocytosis, *AAV* ANCA-associated vasculitis

## Infectious complications following anti-CD20 mAbs

Rituximab impairs the immune homeostasis not only by depleting B cells, but also by disrupting B- and T-cell cross-talk. Together with neutropenia and agranulocytosis, it poses a significant risk of infections not limited to bacteria, but also viruses and opportunistic organisms. Having said that, it is difficult to measure the risk of infections caused by rituximab treatment alone because of the difference in underlying conditions and the immunosuppressive effects of other concomitant therapeutic agents. Overall, about 5% of patients develop infective complications, of which 50% require hospitalisation [32]. The incidence does not appear to increase after repeated courses or higher cumulative doses of rituximab [32]. About 80% of these infections are bacterial, with respiratory infections being the commonest presentation [32–35]. Evidence indicates that rituximab disrupts B-cell and T-cell interactions, which may also increase the risk of certain viral and fungal infections [34]. Pneumocystis Jirovecii pneumonia (PJP) is uncommon but a serious infection that was reported despite the use of antimicrobial prophylaxis [32, 36]. There is an increased risk of hepatitis B virus (HBV) reactivation following treatment with rituximab [37]. Cytomegalovirus (CMV), Epstein–Barr virus (EBV), and BK virus infections may occur in kidney transplant recipients, particularly following treatments for AMR. Importantly, attributing these infections solely to rituximab is challenging, given the frequent use of concurrent immunosuppressive therapies such as corticosteroids and plasma exchange in this setting.

In contrast, reactivations of hepatitis C and tuberculosis are reported less frequently [38–40]. Progressive multifocal leukoencephalopathy (PML), a rare demyelinating disease of the brain caused by JC polyoma virus, was reported in adult patients with rheumatoid arthritis (RA), granulomatosis with polyangiitis (GPA), and microscopic polyangiitis (MPA) treated with rituximab-based chemotherapy, yet the risk is low [41]. Infections appear to be more prevalent in the first 6 months after rituximab administration [22], and interventions are more likely to be required before B cells repopulate [7].

On the other hand, the safety data of obinutuzumab are limited. In the largest available cohort, Dossier et al. described that only one out of the 41 children (2.44%) treated with obinutuzumab for SDNS was hospitalised for pneumonia, with a favourable outcome [42]. Nonetheless, clinicians should consider the use of obinutuzumab more cautiously, due to the longer B-cell depletion period and higher rates of neutropenia and low IgM compared to rituximab [42]. Reports of infection-related adverse events associated with the use of anti-CD20 mAbs in paediatric kidney disease from prospective studies are presented in Table 2. Corresponding data for the adult population are provided in Supplementary Table 2.

**Table 2:** Reports of adverse events related to infection after anti-CD20 use in kidney disease in children from prospective studies

Study	Study Design	Disease	Anti-CD20	No. of Patients Analysed	No. of anti-CD20 courses	Regimen per Course mg/m^2^	Infections, episodes		Serious Infections, episodes		Upper respiratory tract infection
							anti-CD20	Control	anti-CD20	Control	anti-CD20
Brogan *et al.,* 2022 [43]	Prospective	AAV	Rituximab	25	25	1500	105	-	9	-	41
Guigonis *et al., *2008 [44]	Prospective	SDNS and SRNS	Rituximab	22	40	375–1500	NR	-	2	-	NR
Kamei *et al., *2009 [45]	Prospective	Refractory SDNS	Rituximab	12	22	375–1500	NR	-	NR	-	NR
Ravani *et al., *2011 [46]	RCT	NS	Rituximab	54	27	375–750	NR	NR	NR	NR	NR
Magnasco *et al., *2012 [47]	RCT	NS	Rituximab	31	16	750	NR	NR	NR	NR	NR
Ravani *et al., *2013 [48]	Prospective	NS	Rituximab	46	104	375–750	NR	-	3	-	NR
Iijima *et al., *2014 [7]	DBRCT	FRNS and SDNS	Rituximab	48	24	1500	105	42	4^a^	0	NR
Ruggenenti *et al., *2014 [5]	Prospective	FRNS and SDNS	Rituximab	10	10	375–750	NR	-	0	-	0^c^
Sun *et al., *2014 [49]	Prospective	SDNS, FRNS, and SRNS	Rituximab	12	12	375–750	3	-	0	-	2
Ravani *et al., *2015 [8]	RCT	SDNS	Rituximab	30	15	375	NR	NR	NR	NR	NR
Ahn *et al., *2018 [50]	RCT	SDNS	Rituximab	51	35	375–750	21	4	NR	NR	NR
	Prospective	SRNS	Rituximab	23	23	375–750	16	-	NR	-	NR
Basu *et al., *2018 [51]	RCT	CDNS	Rituximab	120	60	750–1500	13^b^	26^b^	2	2	4^b^
Takahashi *et al., *2019 [52]	Prospective	FRNS and SDNS	Rituximab	22	22	1500	25	-	1	-	8
Kari *et al., *2020 [53]	Prospective	SSNS	Rituximab	46	19	750	NR	NR	NR	NR	NR
Ravani *et al., *2020 [54]	RCT	SDNS	Rituximab	30	15	375	NR	NR	NR	NR	NR
Ravani *et al., *2020 [55]	RCT	MRNS	Ofatumumab	13	7	1500mg/1.73 m^2^	NR	NR	NR	NR	NR
Ravani *et al., *2021 [56]	RCT	SDNS	Ofatumumab	140	140	375	NR	NR	NR	NR	NR
Ravani *et al., *2021 [57]	RCT	SDNS	Rituximab	30	15	375	NR	NR	NR	NR	NR
Al Salloum *et al., *2022 [58]	Prospective	SDNS	Rituximab	17	23	750–1500	NR	-	1	-	NR
Mathew *et al., *2022 [59]	RCT	SSNS	Rituximab	41	21	750	86	67	2	2	64
Wang *et al., *2022 [60]	3-arm RCT	FRSDNS	Rituximab	51	17	562.5–750.5	19	71	NR	NR	NR
Basu *et al., *2023 [61]	Prospective	SDNS	Rituximab	119	89	750	NR	NR	NR	NR	NR
Zhu *et al., *2023 [62]	RCT	FRNS and SDNS	Rituximab	29	29	775–1500	17	-	NR	-	NR
Cravedi *et al., *2024 [63]	Prospective	SDNS	Rituximab	13	13	375	0	-	0	-	0
Liu *et al., *2024 [64]	Prospective	SSNS	Rituximab	76	43	375	15^b^	NR	4	NR	10^b^
Nozu *et al., *2024 [65]	Prospective	MRNS	Rituximab	6	6	1500	8	-	2	-	NR
Sheng *et al., *2025 [66]	RCT	NS	Rituximab	24	12	1500	NR	NR	0	0	2^a^
Sinha *et al., *2025 [67]	RCT	FRNS and SDNS	Rituximab	91	91	375–750	NR	-	5	-	NR
Billing *et al., *2008 [68]	Prospective	AMR	Rituximab	6	6	375	0	-	0	-	0
Zarkhin *et al., *2008 [69]	RCT	AMR	Rituximab	20	10	1500	NR	NR	NR	NR	NR
Billing *et al., *2012 [70]	Prospective	AMR	Rituximab	20	20	375	NR	-	1	-	NR
Total				1278	1011		405/233	184/94	32/508	4/116	115/99
Episodes per rituximab course							1.74	1.96	0.06	0.03	1.16

Study	Upper respiratory tract infection	Lower respiratory tract infection		Urinary tract infection		Herpes simplex virus related		Gastrointestinal infection		Sepsis/bloodstream infection	
	Control	anti-CD20	Control	anti-CD20	Control	anti-CD20	Control	anti-CD20	Control	anti-CD20	Control
Brogan *et al.,* 2022 [43]	-	5	-	3	-	NR	-	3	-	1	-
Guigonis *et al., *2008 [44]	-	1	-	NR	-	NR	-	1	-	NR	-
Kamei *et al., *2009 [45]	-	NR	-	NR	-	NR	-	NR	-	NR	-
Ravani *et al., *2011 [46]	NR	NR	NR	NR	NR	NR	NR	NR	NR	NR	NR
Magnasco *et al., *2012 [47]	NR	NR	NR	NR	NR	NR	NR	NR	NR	NR	NR
Ravani *et al., *2013 [48]	-	NR	-	1^ac^	-	NR	-	NR	-	NR	-
Iijima *et al., *2014 [7]	NR	NR	NR	1^ac^	0^ac^	NR	NR	1^ac^	0^ac^	NR	NR
Ruggenenti *et al., *2014 [5]	-	NR	-	NR	-	NR	-	0c	-	NR	-
Sun *et al., *2014 [49]	-	1	-	NR	-	NR	-	NR	-	NR	-
Ravani *et al., *2015 [8]	NR	NR	NR	NR	NR	NR	NR	NR	NR	NR	NR
Ahn *et al., *2018 [50]	NR	NR	NR	NR	NR	NR	NR	NR	NR	NR	NR
	-	NR	-	NR	-	NR	-	NR	-	NR	-
Basu *et al., *2018 [51]	6^b^	4^b^	8^b^	0^b^	2^b^	1^b^	0^b^	3^b^	5^b^	NR	NR
Takahashi *et al., *2019 [52]	-	1	-	NR	-	NR	-	NR	-	NR	-
Kari *et al., *2020 [53]	NR	NR	NR	NR	NR	NR	NR	NR	NR	NR	NR
Ravani *et al., *2020 [54]	NR	NR	NR	NR	NR	NR	NR	NR	NR	NR	NR
Ravani *et al., *2020 [55]	NR	NR	NR	NR	NR	NR	NR	NR	NR	NR	NR
Ravani *et al., *2021 [56]	NR	NR	NR	NR	NR	NR	NR	NR	NR	NR	NR
Ravani *et al., *2021 [57]	NR	NR	NR	NR	NR	NR	NR	NR	NR	NR	NR
Al Salloum *et al., *2022 [58]	-	1	-	NR	-	NR	-	NR	-	NR	-
Mathew *et al., *2022 [59]	47	1	2	NR	NR	NR	NR	5	0	NR	NR
Wang *et al., *2022 [60]	NR	NR	NR	NR	NR	NR	NR	NR	NR	NR	NR
Basu *et al., *2023 [61]	NR	NR	NR	NR	NR	NR	NR	NR	NR	NR	NR
Zhu *et al., *2023 [62]	-	NR	-	NR	-	NR	-	NR	-	NR	-
Cravedi *et al., *2024 [63]	-	0	-	0	-	0	-	0	-	0	-
Liu *et al., *2024 [64]	NR	5^b^	NR	NR	NR	NR	NR	NR	NR	NR	NR
Nozu *et al., *2024 [65]	-	NR	-	NR	-	NR	-	NR	-	NR	-
Sheng *et al., *2025 [66]	0	NR	NR	NR	NR	NR	NR	NR	NR	NR	NR
Sinha *et al., *2025 [67]	-	NR	-	1	-	NR	-	NR	-	1	-
Billing *et al., *2008 [68]	-	0	-	0	-	0	-	0	-	0	-
Zarkhin *et al., *2008 [69]	NR	NR	NR	NR	NR	NR	NR	NR	NR	NR	NR
Billing *et al., *2012 [70]	-	1	-	NR	-	NR	-	NR	-	NR	-
Total	47/32	11/182	2/20	4/135	-	0/19	-	9/105	0/20	2/135	-
Episodes per rituximab course	1.47	0.06	0.10	0.03	-	0.00	-	0.09	0.00	0.01	-

Only AEs without grade scale were included for calculation. No case related to varicella zoster virus, hepatitis B reactivation, cytomegalovirus, Epstein-Barr virus, BK virus, and death due to infection was reported from prospective studies. Therefore, the data of them are not shown

*RCT* randomised controlled trials, *AAV* ANCA-associated vasculitis, *SDNS* steroid dependent nephrotic syndrome, *SRNS* steroid resistant nephrotic syndrome, *FRNS* frequently relapse nephrotic syndrome, *MRNS* multiple drug-resistant nephrotic syndrome, *FRSDNS* frequently-relapsing steroid-dependent nephrotic syndrome, *NR* not reported

^a^Reported by the number of subjects

^b^Grades 2–4

^c^Grades 3–4

## Non-infectious complications mimicking infection: rituximab-induced interstitial lung disease (RTX-ILD)

Rituximab-induced interstitial lung disease (RTX-ILD) is a non-infectious pulmonary complication that can mimic pneumonia clinically. The reported incidence of RTX-ILD is heterogeneous across studies, ranging from 0.01% to 10.0% [71]. Symptoms may occur shortly after rituximab administration, including dyspnoea, cough, and fatigue [72, 73]. The condition is typically resistant to antimicrobials, but can be treated with corticosteroids. In severe cases, patients may progress to fatal respiratory failure, and consequently a high index of suspicion is warranted [72].

## Risk factors for infections

Several factors confound the effects of anti-CD20 mAbs and further increase the risk of infection in children, including neutropenia, hypogammaglobulinaemia, and concomitant use of other immunosuppressive agents. In addition, the nature of the primary disease, such as nephrotic syndrome, and the development of chronic kidney disease may also predispose children to infections [74, 75]. Notably, up to 25% of patients treated with rituximab for severe lupus nephritis developed infections, highlighting the implication of underlying disease on infection [76, 77]. Younger children are more susceptible to developing agranulocytosis and hypogammaglobulinaemia after the use of anti-CD20 mAbs [32, 78]. Early identification and modification of these risk factors may serve as an important strategy to prevent infection.

### Neutropenia and agranulocytosis

Anti-CD20 mAbs result in bone marrow suppression and impair neutrophil production [22, 78, 79]. Neutropenia was described in 1.7%–41.9% and 0.6%–66.7% of children and adults respectively, while agranulocytosis was reported in 2.3%–8.3% of children after rituximab (summarised in Supplementary Tables 3 and 4). In addition, younger age at rituximab is an important associated factor of agranulocytosis [78]. Theoretically, children with neutropenia are susceptible to various types of infections, yet a significant proportion of them developed febrile neutropenia without an identified causative microorganism in a cohort by Chan et al. [32].

### Hypogammaglobulinaemia

The reported rates of hypogammaglobulinaemia are heterogeneous, owing to discrepant local monitoring policies [80]. Even among prospective studies on NS, AAV, and AMR, hypogammaglobulinaemia was reported in 2.3%–91.7% of children and 3.0%–42.4% of adults (Table 3 and Supplementary Table 5). Persistent hypogammaglobulinaemia beyond 1 year post-rituximab is common, and up to 41% of children continue to experience low IgG levels 2 years after the last anti-CD20 administration [81]. In addition to young age, pre-existing hypogammaglobulinaemia prior to anti-CD20 mAbs (e.g. in SRNS with persistent nephrotic-range proteinuria) and concurrent use of immunosuppressants predispose patients to develop and/or perpetuate hypogammaglobulinaemia [24, 32, 81–85]. The significance of hypogammaglobulinaemia on infection risk remains controversial, as only a minor proportion of hypogammaglobinaemia episodes (~ 1%) eventually complicate with significant infections [32, 81, 86]. Clinical studies also observed and reported low IgM and IgA levels [43, 45, 56, 69], yet their roles in preventing infection are inconclusive [87, 88].

**Table 3:** Reported rates of hypogammaglobulinemia after rituximab in children from prospective studies

Study	No. of patients analysed	No. of rituximab courses	Regimen per course mg/m^2^	Outcome measures		Rate of Hypogammaglobulinemia			Serious infectious episodes
						Overall	Baseline IgG levels		
							Normal	Reduced	
**ANCA-associated vasculitis**									
Brogan et al., 2022 [43]	25	25	1500	Patient based	Low IgG at 18 months	11/23 (47.8%)	NR	NR	9
**Childhood nephrotic syndrome**									
Guigonis et al., 2008 [44]	22	40	375–1500	Patient based	Low IgG at last follow-up^a^	8/22 (36.4%)	4/18 (22.2%)	4/4 (100%)	2
Sun et al., 2014 [49]	12	12	375–750	Patient based	Low IgG during rituximab	1/12 (8.3%)	NR	NR	0
Ravani et al., 2020 [54]	30	15	375	Patient based	Low IgG at 6–24 months	0 (0%)	0 (0%)	0 (0%)	NR
Ravani et al., 2021 [57]	30	15	375	Patient based	Low IgG at last follow-up^b^	8/15 (53.3%)	2/6 (33.3%)	6/9 (67%)	0
Al Salloum et al., 2022 [58]	17	23	750–1500	Patient based	Low IgG at 3 years	1/17 (5.9%)	NR	NR	1
Zhu et al., 2023 [62]	29	29	775–1500	Dose based	Low IgG during rituximab	40/95 (42.1%)	NR	NR	0
Cravedi et al., 2024 SC [63]	13	13	375	Patient based	Low IgG at 12 months	0 (0%)	0 (0%)	0 (0%)	0
Liu et al., 2024 [64]	76	43	375	Patient based	Low IgG at disease onset	1 (2.3%)	NR	1/1 (100%)	4
Nozu et al., 2024 [65]	6	6	1500	Patient based	Low IgG at last follow-up^c^	0 (0%)	0 (0%)	0 (0%)	2
Sheng et al., 2025 [66]	24	12	1500	Patient based	Low IgG during rituximab	11/12 (91.7%)	0 (0%)	11/11 (100%)	0
Sinha et al., 2025 [67]	91	91	375–750	Patient based	Low IgG at 18 months	13/91 (14.3%)	NR	NR	6^*^
**Antibody-mediated rejection**									
Billing et al., 2008 [68]	20	20	375	Patient based	Low IgG at 24 months	6/20 (30.0%)	NR	NR	NR

*SC* rituximab subcutaneous injection, *NR* not reported

^*^Number of patients

^a^Median follow-up: 9.5 months

^b^Unpublished data provided upon request by investigators; at last follow-up (median follow-up 286 days)

^c^Persistent hypogammaglobulinemia; 1 followed during rituximab, 5 followed for 2.5 years

Approximately 80% of children relapse after rituximab therapy and require repeated courses to maintain long-term disease control [89]. Multiple treatment courses raise concerns about the development of drug resistance and the potential impact on the long-term immunological profile of a growing child. Colucci et al. studied 27 children with NS treated with rituximab, whose follow-up exceeded 4 years from the first dose and at least 2 years from the last anti-CD20 administration [81]. At last follow-up, total, transitional and mature-naïve B cells had normalised in nearly all patients. In contrast, total memory B cells and switched memory B cells remained significantly reduced in 74% and 78% of cases respectively. IgG levels against hepatitis B virus and tetanus were also further reduced. Although revaccination could induce antigen-specific memory B cells, IgG titres remained low. Similarly, we reported lower antibody seropositivity following COVID-19 vaccination among children receiving rituximab for glomerular disease [90, 91]. More importantly, functional humoral response, measured by surrogate viral neutralisation test, was also impaired after rituximab therapy [90, 91]. Therefore, the use of anti-CD20 should be carefully considered in children during periods of active disease, which often coincide with the critical stage of immune development.

### Concomitant use of immunosuppressants

In SDNS, maintenance immunosuppression with MMF is a well-studied strategy to extend relapse-free remission after anti-CD20 mAbs [89, 92]. It weakens T-cell immunity [85, 93, 94] and predisposes patients to neutropenia, which could potentiate the risk of infections. Iijima et al*.* reported that children receiving maintenance MMF after rituximab had a high rate of neutropenia (12.8% vs 5.1%) and agranulocytosis (5.1% vs 0%), and they were also more susceptible to infection compared to placebo (1.59 vs 0.82 episodes of infections per patient) [92]. Hogan et al. also reported a high rate of VZV infection (25%) when rituximab was used in combination with MMF in paediatric LN patients [95]. In contrast, the relationship between infection and concomitant immunosuppression could not be established in adults treated with rituximab for glomerular disease (MMF, steroids, CNI and cyclophosphamide), except azathioprine and therapeutic plasma exchange [33, 96]. For kidney transplant recipients, the combination of anti-thymocyte globulin and rituximab is associated with a high risk of infection [97, 98].

## Prophylactic measures for patients on anti-CD20 mAbs

The strategies for preventing infection in children receiving anti-CD20 mAbs should extend beyond antimicrobial prophylaxis to include a comprehensive assessment of infection risk, vaccination, and the modification of risk factors.

### Evaluation of infection risk prior to commencement of anti-CD20 mAbs

We recommend evaluating patients for infection risk before initiating anti-CD20 mAbs. A comprehensive history taking (including birth history, past infections, vaccination history and family history) and a thorough physical examination should be undertaken for all children contemplated for anti-CD20 mAbs. This information would guide the subsequent investigations and actions as summarised in Table 4.

**Table 4:** Baseline evaluations before anti-CD20 treatment to assess infective risk

1. General assessment	• Review vaccination record • Review past medical history • Review concurrent drug history • Review travel history
2. Investigations	• Complete blood count • Immunoglobulin G, A, M • G6PD status (for use of cotrimoxazole as PJP prophylaxis) • Screening for Mycobacterium tuberculosis infection - Tuberculin skin test (TST) - Interferon gamma release assay (IGRA) • Serologic testing for (a) Hepatitis B virus (HBV) - Hepatitis B core antibody total (anti-HBc total) - Hepatitis B surface antibody (anti-HBs) - Hepatitis B virus surface antigen (HbsAg) - HBV DNA in HBsAg-positive patients (b) Hepatitis C virus (anti-HCV IgG) (c) Cytomegalovirus (CMV IgG) (d) Epstein-Barr virus (EBV IgG) (e) Human Immunodeficiency Virus (fourth-generation antigen/antibody combination test) (f) Varicella-zoster virus (VZV IgG) (g) Measles, mumps, rubella, if available • Optional: - B-cell subsets - Chest radiograph (to rule out active infection or in case of positive TB screening in high prevalence areas)

### Monitoring following anti-CD20 mAbs

In our practice, we regularly monitor patients’ complete blood count and immunoglobulin G, A, and M levels 1 month after anti-CD20 mAbs administration, and every three months thereafter. While the neutropenia usually occurs early after drug exposure, hypogammaglobulinaemia may perpetuate. Consequently, we monitor immunoglobulin levels for at least 18 months and until the levels normalise. In addition, we would evaluate for neutropenia and hypogammaglobulinaemia during active infection, where active interventions may be required.

### Immunisations

Ideally, children should receive all necessary immunisations prior to anti-CD20 mAbs for optimal immunogenicity, yet it is usually not feasible in most clinical scenarios due to the urgency of treatment. All vaccinations should be given at least 2 to 4 weeks prior to the administration of anti-CD20 mAbs for immunologic response to develop [99, 100]. This is highlighted by the observation that none of the patients achieved seroconversion if hepatitis B vaccine was given within 3 days before rituximab administration [101]. For patients who were given anti-CD20 mAbs during an ongoing vaccine series, seroconversion rates declined with fewer vaccine doses prior to therapy initiation, from 92.8% (95% CI 87.1–96.5) after 4 doses to merely 24.0% (95% CI 9.4–45.1) after 1 dose [102]. Available data suggest that circulating B-cell levels at the time of vaccination correlate with the antibody levels [103–105]. Thus, delaying vaccinations until B-cell reconstitution is a plausible approach to optimise vaccine responses. Pre-existing antibodies from primary immunisation were preserved in some cases treated with rituximab, since the long-lived plasma cells are theoretically unaffected by anti-CD20 mAbs [103, 106, 107]. However, other studies also reported reduced antibody titres following treatment [101, 106]. Higher or additional doses of vaccines, and adjuvanted vaccines, have been used in influenza and SARS-CoV-2 mRNA vaccines to improve immunogenicity in immunocompromised patients [90, 108]. Additional doses of SARS-CoV-2 mRNA vaccines are able to boost antibody responses following rituximab, although the attained antibody titres remained lower compared to children receiving dialysis and healthy controls [90, 109].

The use of inactivated vaccines during or after recent anti-CD20 mAbs is considered safe [110]. In general, inactivated vaccines should be administered at least 5 to 6 months after the last anti-CD20 administration and 2 to 4 weeks prior to the subsequent dose [107, 111]. In addition to diminished vaccine competency, live attenuated vaccines (e.g. measles-mumps-rubella and varicella vaccines) are contraindicated during anti-CD20 mAbs administration owing to the risk of developing severe vaccine-related infection [100]. Although these vaccines have been safely administered to selected patients on long-term immunosuppressants with preserved cellular and humoral immunity [112], such practice has not been formally evaluated in rituximab-treated patients. The desired timing of live-attenuated vaccines is 12 or more months after rituximab, after B-cells repopulate while the patient is not receiving concurrent immunosuppression [107]. In situations where immunisation is not feasible, close monitoring and patient education on vaccine-preventable diseases are essential for timely diagnosis and post-exposure prophylaxis. Vaccination of household and close contacts should be encouraged to protect immunocompromised patients [100]. In summary, clinicians should review immunisation status at diagnosis and, where feasible, timely vaccinate the patients before initiating anti-CD20 mAbs. In glomerular disease, timely vaccination is often challenging due to the need for early immunosuppression. Once anti-CD20 mAbs are administered, vaccination should ideally be deferred until B-cell repopulation to optimise immunogenicity while balancing infection risk. For children progressing rapidly to kidney failure and awaiting a kidney transplant, there is limited data on live vaccines to support the best approach. Inactivated vaccines with post-vaccination antibody titre monitoring may be considered to ensure adequate immune response. Table 5 summarises vaccination recommendations for patients receiving anti-CD20 mAbs.

**Table 5:** Specific recommendations for the vaccines in patients treated with rituximab and other anti-CD20 mAbs [107, 111]

Vaccine	Vaccine type	Suggested dose	Suggested timing of vaccination in relation to rituximab	Serological testing post immunisation
Diphtheria, tetanus, polio vaccine	Inactivated	Usual dose and schedule	Preferably 6 months after last infusion and at least 2 weeks prior to next infusion	Not available in routine setting
Haemophilus conjugate vaccine	Inactivated	Usual dose and schedule	Preferably 6 months after last infusion and at least 2 weeks prior to next infusion	Not available in routine setting
Hepatitis B	Inactivated	Higher dose for immunocompromised people: Double dose of age should be given, total 4 doses (months 0, 1, 2, 6–12) [113]	Preferably 6 months after last infusion and at least 2 weeks prior to next infusion Consider to delay the entire vaccine series until completion of rituximab courses	Anti-HBs at 1–2 months following completion of the vaccine series [100]
Human papillomavirus vaccine	Inactivated	Three doses for immunocompromised people [114] Months 0, 2, 6 from 9 years old onwards	Preferably 6 months after last infusion and at least 2 weeks prior to next infusion Consider delaying the entire vaccine series until completion of rituximab courses	Not available in routine setting
Inactivated influenza Vaccine	Inactivated	Usual dose and schedule Second dose is required for children aged < 9 years who have not previously been vaccinated	Preferably 6 months after last infusion and at least 2 weeks prior to next infusion Can receive during influenza seasons without delay to reduce complications of infections	Not available in routine setting
Measles, rubella, mumps (MMR)	Live-attenuated	Usual dose and schedule	Preferably 12 months after last infusion and 4 weeks prior to next infusion [107]	Measles, mumps, rubella IgG antibodies
Meningococcal ACWY vaccine	Inactivated	Usual dose and schedule	Preferably 6 months after last infusion and at least 2 weeks prior to next infusion	Not available in routine setting
Meningococcal B vaccine	Inactivated	Usual dose and schedule	Preferably 6 months after last infusion and at least 2 weeks prior to next infusion	Not available in routine setting
Pneumococcal 23-valent (PPSV-23)	Inactivated	Usual dose and schedule	Preferably 6 months after last infusion and at least 2 weeks prior to next infusion	Not available in routine setting
Pneumococcal vaccine 15-valent (PCV-15)	Inactivated	Usual dose and schedule	Preferably 6 months after last infusion and at least 2 weeks prior to next infusion	Not available in routine setting
Pneumococcal vaccine 20-valent (PCV-20)	Inactivated	Usual dose and schedule	Preferably 6 months after last infusion and at least 2 weeks prior to next infusion	Not available in routine setting
SARS-CoV2-mRNA vaccine	mRNA	Additional doses for immunocompromised people, please refer to latest CDC recommendation [115, 116]	Preferably 6 months after last infusion and at least 2 weeks prior to next infusion Can receive during peak seasons without delay to reduce complications of infections	Not available in routine setting
Varicella	Live-attenuated	Usual dose and schedule	Preferably 12 months after last infusion and 4 weeks prior to next infusion [107]	Varicella IgG antibodies

### Immunoglobulin replacement

Immune globulin preparations are highly purified IgG antibodies extracted from pooled plasma of qualified donors using methods that vary by manufacturer [117]. Both IVIG and subcutaneous immune globulins (SCIG) have been used in primary immunodeficiencies to reduce infections and associated complications [117]. The policy of prophylactic immune globulin replacement for post-rituximab hypogammaglobulinaemia has been adopted in some centres [118], since these patients are considered to have secondary immunodeficiency and are at risk of infections. In a large cohort study comprising 8633 adults (mean age 59.8 years), higher cumulative immunoglobulin replacement dose reduced the risk of severe infection by 2% (HR 0.98; 95% CI, 0.96–0.99; *P* = 0.002) [21]. Direct data extrapolation to children may not be appropriate and this measure has not been evaluated in paediatric trials. On the other hand, many patients with post-rituximab hypogammaglobulinaemia remain asymptomatic, even with very low levels of IgG [24]. IVIG preparation has a short half-life between 32 and 36 days, and therefore monthly infusion is required, resulting in repeated hospitalisations and compromised quality of life [24]. SCIG is a convenient alternative to IVIG, which allows self-administration at home after adequate training [119]. The decision of regular IVIG infusions should be made jointly with the patient and the family, balancing the potential benefits and drawbacks (Table 6). On the other hand, IgM deficiency is common after rituximab and obinutuzumab, which is a potential risk factor of severe and/or recurrent infections [42]. The use of IgM-enriched immunoglobulin, rather than IVIG or SCIG with minimal content of IgA and IgM, has been successful as an adjunctive treatment of sepsis. However, it has not been studied in detail in the context of infection prophylaxis after anti-CD20 therapy [120].

**Table 6:** Comparisons of immunoglobulin replacement therapy

	Intravenous immunoglobulin	Subcutaneous immunoglobulin
Brand examples	Privigen®, Intragam Nexgen®	Hizentra®, Cuvitru®
Concentration	10%	20%
Route of administration	Intravenous	Subcutaneous
Frequency of administration	Every 4 weeks	Every 1–2 weeks
Duration of infusion	Shorter	Longer
Systemic adverse reactions	Higher	Fewer
Need for hospital visit	Yes, require medical personnel	No, self-administration at home
Need for patient and family training	No	Yes

In adult patients with AAV receiving maintenance rituximab therapy, current recommendations suggest replacing immunoglobulin intravenously at 0.4 g per kg every month if the IgG level is < 300 mg/dL with recurrent severe infections [121]. Some guidelines suggest keeping a higher level of trough IgG level in case of one or more life-threatening infections over the previous 12 months [122]. The optimal target of IgG level remains unclear [123]. An IgG level of 800 mg/dL or lower normal limit for age has been suggested to be the initial replacement target [122, 123], with monthly titration to achieve infection-free status. IVIG is generally well tolerated but can also cause adverse reactions such as infusion reactions, haemolysis and thromboembolism [124]. Of note, acute kidney injury may follow exposure to sucrose-containing immune globulin preparation, especially among patients with pre-existing kidney impairment [125]. The risk may be mitigated by pre- and post-hydration and limiting the infusion rate.

### Antimicrobial prophylaxis and specific considerations

Antimicrobial prophylaxis should be personalised alongside standard preventive measures. Several factors warrant careful evaluation, including the patient’s risk factors, baseline evaluation results, the type, severity, and frequency of previous infections, as well as the costs and potential side effects of antimicrobials [75, 126]. Generally, antimicrobial prophylaxis may be considered for patients with persistent hypogammaglobulinaemia and recurrent infections, tailored to the potential pathogens based on their infection history [21]. Prophylaxis against several microorganisms and viruses is detailed in the following section and Table 7.

**Table 7:** Available options of prophylaxis to specific infections [117]

Infection	Prophylaxis regimen	Renal dose adjustment	Adverse reactions	Duration of prophylaxis and monitoring
Cytomegalovirus (CMV) [127, 128]	Valganciclovir PO < 16 years old: Daily dose = 7 × BSA (m^2^) × eGFR; Max 450 mg for intermediate-risk transplant recipient (donor CMV +ve to recipient CMV +ve); and Max 900 mg for high-risk transplant recipient (donor CMV +ve to recipient CMV −ve)	eGFR < 60; paediatric formula has incorporated eGFR in dose calculation	Neutropenia, nausea, diarrhoea, anaemia, hypertension	High-risk transplant recipient: 6 months Intermediate-risk transplant recipient: 3 months
Herpes simplex virus (HSV) [75]	*Prevention for recurrent infections* Acyclovir 80 mg/kg/day in 2 to 3 divided doses PO, max 800 mg per dose	eGFR < 25	Acute kidney failure, neutropenia, anaemia, thrombocytopenia	Until at least 6 months after last rituximab infusion
	*Prevention for recurrent infections* Valacyclovir 20 mg/kg (max 500 mg) BD PO	eGFR < 30	Abdominal pain, nausea, headache, nasopharyngitis, acute kidney failure, neutropenia,	
Hepatitis B [129]	*HBV reactivation* Entecavir > 2 years old: 0.015 mg/kg (max 0.5 mg) daily PO	eGFR < 50	Increased transaminases, glycosuria, haematuria	Until 12–18 months after last rituximab infusion, followed by 12 months of close surveillance after cessation of prophylaxis
	Tenofovir disoproxil fumarate > 2 years old: 8 mg/kg (max 300 mg) daily PO	eGFR < 50	Nephrotoxicity, decreased bone mineral density	
	Tenofovir alafenamide > 6 years old: 25 mg daily PO	eGFR < 30	Headache, increased transaminases	
	*Post-exposure in anti-HBs negative patients* Hepatitis B hyperimmune globulin (HBIG) 0.06 mL/kg (max 5 mL) IM as soon as possible after exposure (within 24 h of needlestick, ocular or mucosal exposure) Consider giving a second dose of HBIG 4 weeks later (due to suboptimal vaccine response)	Not required	Headache, malaise, myalgia, pain at injection site, erythema	1–2 doses
Influenza A and B [130]	*Post-exposure prophylaxis* *Oseltamivir* 10–15 kg: 30 mg daily > 15–23 kg: 45 mg daily > 23–40 kg: 60 mg daily > 40 kg: 75 mg daily	eGFR < 60	Nausea, vomiting, headache, skin reactions	7 days
	*Inhaled zanamivir* > 5 years old: 10 mg (two 5 mg inhalation) daily	Not required	Bronchospasm, skin reactions	7 days
Pneumocystis jirovecii [131]	Trimethoprim-sulfamethoxazole (TMP-SMX)/cotrimoxazole (suggested) 2.5–5 mg/kg (max 160 mg) TMP daily or 3 times per week PO	eGFR < 30	Hypersensitivity reactions (rashes, fever), neutropenia, hyperkalaemia, increased transaminases, kidney failure	Until at least 6 months after last rituximab infusion, or until B-cell reconstitution, whichever longer
	Inhaled pentamidine (> 5 years old) 300 mg every month, with nebulised salbutamol as pre-medication IV pentamidine > 2 years old: 4 mg/kg (max 300 mg) every 4 weeks	Not required	Nephrotoxicity, hyper or hypokalaemia, hypoglycaemia, hypocalcaemia, arrhythmias, pancreatitis, respiratory symptoms	
	Dapsone 2 mg/kg (max 100 mg) daily or 4 mg/kg (max 200 mg) weekly PO	Not required	Rash, fever, lymphadenopathy, haemolytic anaemia, increased transaminases, methemoglobinemia, neutropenia	
	Atovaquone > 2 years old: 30 mg/kg (max 1500 mg) daily PO	Not required	Nausea, diarrhoea, fever, hepatitis, rash	
Tuberculosis [132]	*Reactivation of latent infection* (1) Isoniazid monotherapy for 6 or 9 months 10–20 mg/kg (children), 5 mg/kg (adult) (max 300 mg) daily PO (2) Isoniazid and rifapentine combination therapy for 3 months **Isoniazid** 2–11 years old: 25 mg/kg (max 900 mg) once weekly PO > 12 years old: 15 mg/kg (max 900 mg) once weekly PO **Rifapentine** (10–14 kg) 300 mg once weekly PO (14.1–25 kg) 450 mg once weekly PO (25.1–32 kg) 600 mg once weekly PO (32.1–49.9 kg) 750 mg once weekly PO > 50 kg 900 mg once weekly PO (3) Rifampicin monotherapy for 4 months 15–20 mg/kg (children) or 10 mg/kg (adult) (max 600 mg) daily PO (4) Isoniazid and rifampicin combination therapy for 3 months **Isoniazid** 10–20 mg/kg (children), 5 mg/kg (adult) (max 300 mg) daily PO **Rifampicin** 15–20 mg/kg (children) or 10 mg/kg (adult) (max 600 mg) daily PO	Not required	*Isoniazid* Peripheral neuropathy, hepatotoxicity, rash, hypersensitivity *Rifapentine* Anaemia, thrombocytopenia, neutropenia *Rifampicin* Hepatotoxicity, rash or other allergy, thrombocytopenia, gastrointestinal upset	Duration varies with the regimen (as mentioned)
Varicella zoster virus (VZV) [133]	*Post-exposure prophylaxis* Acyclovir 20 mg/kg/dose four times a day (max 800 mg per dose) for 7 days PO, beginning 7 days after exposure	eGFR < 25	Acute kidney failure, neutropenia, anaemia, thrombocytopenia	7 days
	*Post-exposure prophylaxis* Varicella immunoglobulin IM injection (VARIZIG®) 125 IU/10 kg body weight, up to a maximum of 625 IU, ideally within 96 h but can be given up to 10 days after exposure	Not required	Pain at injection site, skin rash	1 dose

## Prophylaxis against specific microorganisms

### Pneumocystis Jirovecii

While clinical practice is heterogeneous and evidence remains limited, in view of the high mortality of pneumocystis Jirovecii (PJP) infection, prophylaxis has been suggested in some centres for patients receiving anti-CD20 mAbs, particularly for those receiving concurrent immunosuppressants with high-dose corticosteroids [9, 15, 126, 134]. Trimethoprim-sulfamethoxazole (TMP-SMX or cotrimoxazole) is the preferred choice for prophylaxis at a dose of 5–10 mg/kg TMP component (max 160 mg TMP/800 mg SMX) daily or three times per week [135]. Similar efficacy between daily and thrice-weekly regimens was observed in childhood leukaemia [136], although some studies favoured a daily dosing regimen [136]. However, adverse effects including neutropenia and skin eruption are common, with higher incidences in daily dosing regimens. Recent evidence in the transplant population suggests that daily low-dose cotrimoxazole (2.5 mg/kg TMP) may provide adequate protection against PJP and is better tolerated [137, 138]. PJP prophylaxis is given for at least 6 months following the last anti-CD20 infusion [75, 131] and should cover the period of B-cell depletion [9]. The duration may be extended in those treated with obinutuzumab, who often experience a longer duration of B-cell depletion [42]. A high index of suspicion is warranted as late-onset PJP infections may occur even after drug discontinuation [139]. In addition, prophylactic use of cotrimoxazole was associated with a lower frequency of severe infections (predominantly respiratory tract infections) (HR 0.30; 95% CI 0.13–0.69) in an adult population with AAV [140]. Pentamidine can be used in case of cotrimoxazole allergy or intolerance and G6PD deficiency (Table 7). Other alternatives include dapsone and atovaquone.

### Hepatitis B and C virus

Use of anti-CD20 mAbs is associated with high risk (> 10%) of HBV reactivation among carriers [141]. For this reason, it is advisable to check both HBsAg and anti-HBc (IgG or total) prior to therapy. High-risk patients, who are HBsAg positive or HBsAg negative/anti-HBc positive, should be additionally assessed by HBV DNA viral load. Current guidelines suggest that antiviral prophylaxis with high barrier to resistance (e.g. entecavir or tenofovir) should be initiated 1 week before or at the time of anti-CD20 mAbs initiation and continued for at least 12–18 months after the last administration [141–144]. Close surveillance for late HBV reactivation is indicated for 12 months following cessation of antiviral prophylaxis [143, 144]. In patients with negative anti-HBs (titre < 10 mIU/mL), due to suboptimal vaccine responses during anti-CD20 mAbs, precautionary measures to avoid HBV exposure are important, and passive immunisation with hepatitis B immunoglobulin can be offered within 48 h following exposure (e.g. contact with HBsAg-positive individuals via blood, mucosal or sexual routes) [113]. Rare cases of rituximab-induced hepatitis C reactivation were reported in lymphoma and rheumatoid arthritis [145]. However, there are no existing guidelines to recommend routine prophylaxis against hepatitis C infection during anti-CD20 mAbs.

### Cytomegalovirus virus (CMV) and Epstein–Barr virus (EBV)

In kidney transplant recipients, CMV reactivation and CMV disease may occur following anti-CD20 therapy for AMR [38]. Therefore, it is recommended to initiate valganciclovir prophylaxis among moderate- and high-risk patients (particularly CMV donor-positive, recipient-negative pair) for 3 to 6 months, with close surveillance (CMV PCR or CMV pp65) for late-onset CMV reactivation after discontinuation of prophylaxis [127]. Notably, valganciclovir may contribute to an additional risk of developing neutropenia with concurrent rituximab administration [146]. In contrast, there is no effective prophylaxis against EBV. Regular surveillance for EBV PCR is advisable in transplant recipients after intensive immunosuppression [147] 

### Influenza infection

For influenza A and B, post-exposure prophylaxis (preferably oseltamivir) is indicated within 48 h of exposure to prevent influenza complications, since anti-CD20 mAbs-treated patients may not have mounted a sufficient immune response to influenza vaccine [130].

### Herpes simplex virus (HSV) and varicella zoster virus (VZV)

HSV and VZV reactivation is a frequent complication following anti-CD20 therapy. In patients with recurrent HSV or VZV infections, valacyclovir or acyclovir prophylaxis can be considered until 6 months after the last dose of anti-CD20 administration [75]. Patients without immunity to varicella (VZV IgG negative) should be offered varicella zoster immunoglobulin within 96 h following exposure [133]. A 7-day course of antiviral (acyclovir or valacyclovir), beginning within 7 and 10 days post-exposure, can be considered if immunoglobulin cannot be administered within the aforementioned timeframe.

### Human immunodeficiency virus (HIV)

In HIV-infected children, use of anti-CD20 mAbs is associated with clinically relevant adverse effects such as leukopenia, neutropenia and transient decline in CD4+ and CD8+ T-cell counts that potentially increase susceptibility to infections [148]. There are no specific recommendations on additional antimicrobial prophylaxis in patients with HIV after anti-CD20 mAbs. Antimicrobial prophylaxis should follow the existing recommendations for HIV disease based on CD4 count and history of infections [149].

### Mycobacterium tuberculosis

Compared to anti-TNF therapies and moderate to high doses of corticosteroids, the risk of tuberculosis (TB) reactivation following anti-CD20 therapy is relatively low. In the context of childhood kidney diseases where rituximab is indicated, most children often receive concurrent immunosuppressive agents. Therefore, TB should be excluded through a thorough medical history and clinical examination in all children. Chest radiograph should be considered in children residing in high-prevalence areas or with a positive travel history. If active TB is diagnosed, anti-tuberculous treatment should be initiated prior to commencing immunosuppressants. For latent TB infection, defined as asymptomatic patients with a positive tuberculin skin test or interferon gamma release assay, appropriate treatment should be offered concurrently [150]. While rifampicin-based regimens are generally safe and associated with high treatment completion rates [132, 151], rifampicin can cause drug-drug interactions and increase the clearance of corticosteroids. Consequently, isoniazid monotherapy for 6–9 months may be considered to minimise potential drug interactions. Patients receiving isoniazid should undergo regular liver function monitoring and take pyridoxine (vitamin B6) throughout the course of treatment to prevent peripheral neuropathy [152].

## Conclusion

Although anti-CD20 mAb has become an essential treatment in several immune-mediated childhood kidney diseases, its safety profile in paediatric patients has not been fully established. While immunosuppressed children are at an increased risk of infections, many of these can potentially be prevented or their risks reduced through strategies such as exposure avoidance, vaccination, antimicrobial prophylaxis, and the use of immunoglobulins when indicated. Clinicians can help mitigate the risk of serious infections associated with anti-CD20 mAbs by identifying patients with relevant risk factors and understanding the mechanisms and risks of immunosuppression. Screening and counselling should be performed prior to initiating therapy, and patients with complex exposure histories or latent infections should be referred to infectious disease specialists for further evaluation and management. Continuous risk assessments and education should be provided throughout treatment, especially during disease flares. Last but not least, education of patients and their families regarding non-pharmacological infection prevention strategies, together with vigilant monitoring for signs and symptoms of infection, remains crucial to ensuring safe and effective treatment outcomes (Table 8).

**Table 8:** Practice points for minimising risk of infection for anti-CD20 mAbs in paediatric kidney diseases

Practice Points	
1) Pre therapy assessment and immunisation: o Detailed evaluation on the medical history, review the indications of anti-CD20 mAbs, concurrent use of immunosuppressants, vaccination history, and travel history o Screen for active/latent infections (see Table 4), consult infectious disease specialists for complex cases o Administer necessary vaccines 2–4 weeks before anti-CD20 mAbs 2) If vaccination cannot be completed before anti-CD20 mAbs: o Surveillance for infections o Re-vaccinate at an appropriate interval after the last anti-CD20 mAbs administration, considering B-cell reconstitution 3) After anti-CD20 mAbs: o Adjust and minimise concurrent immunosuppression o Monitor complete blood count for neutropenia and agranulocytosis (may require GCSF treatment) o Antimicrobial prophylaxis (preferably cotrimoxazole if no G6PD deficiency) against Pneumocystis jirovecii pneumonia ≥ 6 months, may be extended until B-cell reconstitution o For children with persistent hypogammaglobulinaemia and recurrent or severe infections, consider prophylactic immune globulin (either intravenous or subcutaneous) o CMV prophylaxis in moderate- to high-risk transplant recipients for 3–6 months o Promote seasonal influenza vaccination (see Table 5 for caution) 4) Comprehensive education of patients and their families o Infection prevention strategies o Monitor for signs and symptoms of infection for early intervention	

## Key summary points

1. Anti-CD20 mAbs are increasingly used in the management of childhood kidney diseases, yet there are concerns about their side effects, especially infection.
2. A structured approach should be adopted to assess and mitigate infection risk in children receiving anti-CD20 mAbs.
3. In addition, monitoring for neutropenia and hypogammaglobulinaemia, antimicrobial prophylaxis, immunisation, and the modification of risk factors should be considered to optimise safety in children receiving anti-CD20 mAbs.
4. Further prospective, large cohort paediatric studies, particularly for new generations of anti-CD20 mAbs, are warranted to explore their infection risks and refine prophylaxis strategies.

## Multiple-choice questions

Answers are provided following the references.

1. In which of the following conditions may anti-CD20 mAbs be indicated?
  A Nephrotic syndrome
  B ANCA-associated vasculitis nephritis
  C Antibody-mediated rejection
  D All of the above
  E None of the above
2. Which of the followings should be screened prior to anti-CD20 mAbs?
  A Immunoglobulin G (A, M)
  B Complete blood count
  C Hepatitis B and C serology
  D Interferon gamma (or tuberculin skin test, if not receiving immunosuppression)
  E All of the above
3. Which of the following is the preferred prophylaxis against pneumocystis Jirovecii?
  A Trimethoprim-sulfamethoxazole
  B Penicillin
  C Pentamidine
  D Dapsone
  E Oseltamivir
4. When should we administer inactivated vaccine after anti-CD20 mAbs?
  A 5 to 6 months after anti-CD20 mAbs
  B 5 to 6 months after anti-CD20 mAbs with B cell repopulation
  C 1 month after anti-CD20 mAbs
  D No restriction
  E 1 year after anti-CD20 mAbs

## Supplementary information

Below is the link to the electronic supplementary material.

ESM 1 (DOCX 68.2 KB)

ESM 2 (XLSX 18.8 KB)

Graphical abstract (PPTX 213 KB)

## References

[1] Lee DSW, Rojas OL, Gommerman JL (2021) B cell depletion therapies in autoimmune disease: advances and mechanistic insights. Nat Rev Drug Discov 20:179–199. 10.1038/s41573-020-00092-2 33324003 10.1038/s41573-020-00092-2 PMC7737718

[2] Plosker GL, Figgitt DP (2003) Rituximab: a review of its use in non-Hodgkin’s lymphoma and chronic lymphocytic leukaemia. Drugs 63:803–843. 10.2165/00003495-200363080-00005 12662126 10.2165/00003495-200363080-00005

[3] Virgolini L, Marzocchi V (2004) Rituximab in autoimmune diseases. Biomed Pharmacother 58:299–309. 10.1016/j.biopha.2004.04.006 15194166 10.1016/j.biopha.2004.04.006

[4] Marlais M, Wlodkowski T, Printza N, Kronsteiner D, Krisam R, Sauer L, Aksenova M, Ashoor I, Awan A, Bacchetta J, Balasubramanian R, Basu B, Bekassy Z, Boyer O, Chan EY, Csaicsich D, Decramer S, Dorresteijn E, Drozynska-Duklas M, Eid LA, Espinosa L, Ferraris V, Flogelova H, Forero-Delgadillo J, Gianviti A, Gracchi V, Gonzalez ML, Hansen M, Hattori M, Hong X, Hooman N, Ivanov D, Kang HG, Karava V, Kazyra I, Lungu A, Marks S, Maxted A, Moczulska A, Muller R, Nastausheva T, Parolin M, Pecoraro C, Principi I, Sanchez-Kazi C, Saygili S, Schild R, Shenoy M, Sinha R, Spizzirri AP, Stack M, Szczepanska M, Tsygin A, Tzeng J, Urbonas V, Zapata C, Zieg J, Schaefer F, Vivarelli M, Tullus K (2023) Clinical factors and adverse kidney outcomes in children with antineutrophil cytoplasmic antibody-associated glomerulonephritis. Am J Kidney Dis 81:119–122. 10.1053/j.ajkd.2022.05.013 35810826 10.1053/j.ajkd.2022.05.013

[5] Ruggenenti P, Ruggiero B, Cravedi P, Vivarelli M, Massella L, Marasà M, Chianca A, Rubis N, Ene-Iordache B, Rudnicki M, Pollastro RM, Capasso G, Pisani A, Pennesi M, Emma F, Remuzzi G (2014) Rituximab in steroid-dependent or frequently relapsing idiopathic nephrotic syndrome. J Am Soc Nephrol 25:850–863. 10.1681/ASN.2013030251 24480824 10.1681/ASN.2013030251 PMC3968490

[6] Benz K, Dötsch J, Rascher W, Stachel D (2004) Change of the course of steroid-dependent nephrotic syndrome after rituximab therapy. Pediatr Nephrol 19:794–797. 10.1007/s00467-004-1434-z 15071769 10.1007/s00467-004-1434-z

[7] Iijima K, Sako M, Nozu K, Mori R, Tuchida N, Kamei K, Miura K, Aya K, Nakanishi K, Ohtomo Y, Takahashi S, Tanaka R, Kaito H, Nakamura H, Ishikura K, Ito S, Ohashi Y, Rituximab for Childhood-onset Refractory Nephrotic Syndrome (RCRNS) Study Group (2014) Rituximab for childhood-onset, complicated, frequently relapsing nephrotic syndrome or steroid-dependent nephrotic syndrome: a multicentre, double-blind, randomised, placebo-controlled trial. Lancet 384:1273–1281. 10.1016/S0140-6736(14)60541-9 24965823 10.1016/S0140-6736(14)60541-9

[8] Ravani P, Rossi R, Bonanni A, Quinn RR, Sica F, Bodria M, Pasini A, Montini G, Edefonti A, Belingheri M, De Giovanni D, Barbano G, Degl’Innocenti L, Scolari F, Murer L, Reiser J, Fornoni A, Ghiggeri GM (2015) Rituximab in children with steroid-dependent nephrotic syndrome: a multicenter, open-label, noninferiority, randomized controlled trial. J Am Soc Nephrol 26:2259–2266. 10.1681/ASN.2014080799 25592855 10.1681/ASN.2014080799 PMC4552120

[9] Trautmann A, Boyer O, Hodson E, Bagga A, Gipson DS, Samuel S, Wetzels J, Alhasan K, Banerjee S, Bhimma R, Bonilla-Felix M, Cano F, Christian M, Hahn D, Kang HG, Nakanishi K, Safouh H, Trachtman H, Xu H, Cook W, Vivarelli M, Haffner D, International Pediatric Nephrology Association (2023) IPNA clinical practice recommendations for the diagnosis and management of children with steroid-sensitive nephrotic syndrome. Pediatr Nephrol 38:877–919. 10.1007/s00467-022-05739-3 36269406 10.1007/s00467-022-05739-3 PMC9589698

[10] Trautmann A, Vivarelli M, Samuel S, Gipson D, Sinha A, Schaefer F, Hui NK, Boyer O, Saleem MA, Feltran L, Müller-Deile J, Becker JU, Cano F, Xu H, Lim YN, Smoyer W, Anochie I, Nakanishi K, Hodson E, Haffner D, International Pediatric Nephrology Association (2020) IPNA clinical practice recommendations for the diagnosis and management of children with steroid-resistant nephrotic syndrome. Pediatr Nephrol 35:1529–1561. 10.1007/s00467-020-04519-1 32382828 10.1007/s00467-020-04519-1 PMC7316686

[11] Floege J, Gibson KL, Vivarelli M, Liew A, Radhakrishnan J, Rovin BH (2025) KDIGO 2025 clinical practice guideline for the management of nephrotic syndrome in children. Kidney Int 107:S241–S289. 10.1016/j.kint.2024.11.007 40254391 10.1016/j.kint.2024.11.007

[12] Chan EY, Boyer O (2025) Childhood idiopathic nephrotic syndrome: recent advancements shaping future guidelines. Pediatr Nephrol 40:2431–2442. 10.1007/s00467-024-06634-9 39724419 10.1007/s00467-024-06634-9 PMC12187818

[13] Kidney Disease: Improving Global Outcomes (KDIGO) Glomerular Diseases Work Group (2021) KDIGO 2021 clinical practice guideline for the management of glomerular diseases. Kidney Int 100:S1–S276. 10.1016/j.kint.2021.05.021 34556256 10.1016/j.kint.2021.05.021

[14] Kidney Disease: Improving Global Outcomes (KDIGO) Glomerular Diseases Work Group (2009) KDIGO clinical practice guideline for the care of kidney transplant recipients. Am J Transplant 9(Suppl 3):S1-155. 10.1111/j.1600-6143.2009.02834.x 10.1111/j.1600-6143.2009.02834.x 19845597

[15] Kidney Disease: Improving Global Outcomes (KDIGO) ANCA Vasculitis Work Group (2024) KDIGO 2024 clinical practice guideline for the management of antineutrophil cytoplasmic antibody (ANCA)-associated vasculitis. Kidney Int 105:S71–S116. 10.1016/j.kint.2023.10.008 10.1016/j.kint.2023.10.008 38388102

[16] Rovin BH, Ayoub IM, Chan TM, Liu Z-H, Mejía-Vilet JM, Floege J (2024) KDIGO 2024 clinical practice guideline for the management of lupus nephritis. Kidney Int 105:S1–S69. 10.1016/j.kint.2023.09.002 38182286 10.1016/j.kint.2023.09.002

[17] Chan EY, Sinha A, Yu ELM, Akhtar N, Angeletti A, Bagga A, Banerjee S, Boyer O, Chan CY, Francis A, Ghiggeri GM, Hamada R, Hari P, Hooman N, Hopf LS, I MI, Ijaz I, Ivanov DD, Kalra S, Kang HG, Lucchetti L, Lugani F, Ma AL, Morello W, Camargo Muniz MD, Pradhan SK, Prikhodina L, Raafat RH, Sinha R, Teo S, Tomari K, Vivarelli M, Webb H, Yap HK, Yap DY, Tullus K (2024) An international, multi-center study evaluated rituximab therapy in childhood steroid-resistant nephrotic syndrome. Kidney Int 106:1146–1157. 10.1016/j.kint.2024.09.011 39395629 10.1016/j.kint.2024.09.011

[18] Chan EY, Marks SD (2025) Childhood-onset lupus nephritis: unique aspects and challenges in management. Kidney Int 108:799–810. 10.1016/j.kint.2025.05.039 40846176 10.1016/j.kint.2025.05.039

[19] Sellier-Leclerc A-L, Belli E, Guérin V, Dorfmüller P, Deschênes G (2013) Fulminant viral myocarditis after rituximab therapy in pediatric nephrotic syndrome. Pediatr Nephrol 28:1875–1879. 10.1007/s00467-013-2485-9 23700173 10.1007/s00467-013-2485-9

[20] Evens AM, Jovanovic BD, Su YC, Raisch DW, Ganger D, Belknap SM, Dai MS, Chiu BC, Fintel B, Cheng Y, Chuang SS, Lee MY, Chen TY, Lin SF, Kuo CY (2011) Rituximab-associated hepatitis B virus (HBV) reactivation in lymphoproliferative diseases: meta-analysis and examination of FDA safety reports. Ann Oncol 22:1170–1180. 10.1093/annonc/mdq58 21115603 10.1093/annonc/mdq583 PMC3082161

[21] Barmettler S, Ong MS, Farmer JR, Choi H, Walter J (2018) Association of immunoglobulin levels, infectious risk, and mortality with rituximab and hypogammaglobulinemia. JAMA Netw Open 1:e184169. 10.1001/jamanetworkopen.2018.4169 30646343 10.1001/jamanetworkopen.2018.4169 PMC6324375

[22] McAtee CL, Lubega J, Underbrink K, Curry K, Msaouel P, Barrow M, Muscal E, Lotze T, Srivaths P, Forbes LR, Allen C, Bernhardt MB (2021) Association of rituximab use with adverse events in children, adolescents, and young adults. JAMA Netw Open 4:e2036321. 10.1001/jamanetworkopen.2020.36321 33533931 10.1001/jamanetworkopen.2020.36321 PMC7859842

[23] Chan EY, Yap DY, Colucci M, Ma AL, Parekh RS, Tullus K (2023) Use of rituximab in childhood idiopathic nephrotic syndrome. Clin J Am Soc Nephrol 18:533–548. 10.2215/CJN.08570722 36456193 10.2215/CJN.08570722 PMC10103321

[24] Chan EY-H, Ma AL-T, Tullus K (2022) Hypogammaglobulinaemia following rituximab therapy in childhood nephrotic syndrome. Pediatr Nephrol 37:927–931. 10.1007/s00467-021-05345-9 34999985 10.1007/s00467-021-05345-9

[25] Zonozi R, Wallace ZS, Laliberte K, Huizenga NR, Rosenthal JM, Rhee EP, Cortazar FB, Niles JL (2021) Incidence, clinical features, and outcomes of late-onset neutropenia from rituximab for autoimmune disease. Arthritis Rheum 73:347–354. 10.1002/art.41501 10.1002/art.41501 PMC7902364 32892495

[26] Otani IM, Ballow M (2025) If and when to consider prophylactic immunoglobulin replacement therapy in secondary hypogammaglobulinemia. J Allergy Clin Immunol Pract 13:511–521. 10.1016/j.jaip.2024.12.024 39725313 10.1016/j.jaip.2024.12.024

[27] Cragg MS, Morgan SM, Chan HTC, Morgan BP, Filatov AV, Johnson PWM, French RR, Glennie MJ (2003) Complement-mediated lysis by anti-CD20 mAb correlates with segregation into lipid rafts. Blood 101:1045–1052. 10.1182/blood-2002-06-1761 12393541 10.1182/blood-2002-06-1761

[28] Lai FF, Chan EY, Tullus K, Ma AL (2024) Therapeutic drug monitoring in childhood idiopathic nephrotic syndrome: a state of the art review. Pediatr Nephrol 39:85–103. 10.1007/s00467-023-05974-2 37147510 10.1007/s00467-023-05974-2

[29] Rossi GM, Baier E, Vaglio A (2025) Obinutuzumab for the management of immune-mediated glomerular diseases. Nephrol Dial Transplant 40:1443–1448. 10.1093/ndt/gfaf021 39900476 10.1093/ndt/gfaf021

[30] Genentech, Inc. (2021) Rituxan (rituximab) prescribing information [package insert]. U.S. Food and Drug Administration. https://www.accessdata.fda.gov/drugsatfda_docs/label/2021/103705s5467lbl.pdf. Accessed 13 Aug 2025

[31] Chan EYH, Lin KYK, Yap DYH, Ma ALT (2025) Obinutuzumab as a viable therapeutic strategy in rituximab-refractory childhood frequently relapsing, steroid-dependent nephrotic syndrome that relapsed during B-cell depletion. Pediatr Nephrol 40:711–714. 10.1007/s00467-024-06570-8 10.1007/s00467-024-06570-8 PMC11753364 39466391

[32] Chan EY, Yu ELM, Angeletti A, Arslan Z, Basu B, Boyer O, Chan CY, Colucci M, Dorval G, Dossier C, Drovandi S, Ghiggeri GM, Gipson DS, Hamada R, Hogan J, Ishikura K, Kamei K, Kemper MJ, Ma AL, Parekh RS, Radhakrishnan S, Saini P, Shen Q, Sinha R, Subun C, Teo S, Vivarelli M, Webb H, Xu H, Yap HK, Tullus K (2022) Long-term efficacy and safety of repeated rituximab to maintain remission in idiopathic childhood nephrotic syndrome: an international study. J Am Soc Nephrol 33:1193–1207. 10.1681/ASN.2021111472 35354600 10.1681/ASN.2021111472 PMC9161790

[33] Trivin C, Tran A, Moulin B, Choukroun G, Gatault P, Courivaud C, Augusto JF, Ficheux M, Vigneau C, Thervet E, Karras A (2017) Infectious complications of a rituximab-based immunosuppressive regimen in patients with glomerular disease. Clin Kidney J 10:461–469. 10.1093/ckj/sfw101 28852482 10.1093/ckj/sfw101 PMC5570029

[34] Nixon A, Ogden L, Woywodt A, Dhaygude A (2017) Infectious complications of rituximab therapy in renal disease. Clin Kidney J 10:455–460. 10.1093/ckj/sfx038 28852481 10.1093/ckj/sfx038 PMC5570071

[35] Heusele M, Clerson P, Guery B, Lambert M, Launay D, Lefevre G, Morell-Dubois S, Maillard H, Le Gouellec N, Hatron PY, Hachulla E (2014) Risk factors for severe bacterial infections in patients with systemic autoimmune diseases receiving rituximab. Clin Rheumatol 33:799–805. 10.1007/s10067-014-2509-2 24487486 10.1007/s10067-014-2509-2 PMC4058071

[36] Sato M, Ito S, Ogura M, Kamei K, Miyairi I, Miyata I, Higuchi M, Matsuoka K (2013) Atypical *Pneumocystis jiroveci* pneumonia with multiple nodular granulomas after rituximab for refractory nephrotic syndrome. Pediatr Nephrol 28:145–149. 10.1007/s00467-012-2286-6 22948319 10.1007/s00467-012-2286-6

[37] Barone M, Notarnicola A, Lopalco G, Viggiani MT, Sebastiani F, Covelli M, Iannone F, Avolio AW, Di Leo A, Cantarini L, Lapadula G (2015) Safety of long-term biologic therapy in rheumatologic patients with a previously resolved hepatitis B viral infection. Hepatology 62:40–46. 10.1002/hep.27716 25613809 10.1002/hep.27716

[38] Los-Arcos I, Len O, Perello M, Torres IB, Codina G, Esperalba J, Sellarés J, Moreso F, Seron D, Gavaldà J (2019) Is antibody-mediated rejection in kidney transplant recipients a risk factor for developing cytomegalovirus or BK virus infection? Results from a case-control study. J Clin Virol 110:45–50. 10.1016/j.jcv.2018.11.010 30537648 10.1016/j.jcv.2018.11.010

[39] Bailly E, Ville S, Blancho G, Morelon E, Bamoulid J, Caillard S, Chatelet V, Malvezzi P, Tourret J, Vuiblet V, Anglicheau D, Bertrand D, Grimbert P, Haidar F, Hazzan M, Kamar N, Merville P, Mousson C, Pernin V, Pouteil-Noble C, Purgus R, Sayegh J, Westeel P-F, Sautenet B, Gatault P, Büchler M (2020) An extension of the RITUX-ERAH study, multicenter randomized clinical trial comparing rituximab to placebo in acute antibody-mediated rejection after renal transplantation. Transpl Int 33:786–795. 10.1111/tri.13613 32279367 10.1111/tri.13613

[40] Naciri Bennani H, Daligault M, Noble J, Bardy B, Motte L, Giovannini D, Emprou C, Fiard G, Imerzoukene F, Bourdin A, Masson D, Janbon B, Malvezzi P, Rostaing L, Jouve T (2021) Treatment of antibody-mediated rejection with double-filtration plasmapheresis, low dose IVIg plus rituximab after kidney transplantation. J Clin Apher 36:584–594. 10.1002/jca.21897 33783868 10.1002/jca.21897

[41] Berger JR, Malik V, Lacey S, Brunetta P, Lehane PB (2018) Progressive multifocal leukoencephalopathy in rituximab-treated rheumatic diseases: a rare event. J Neurovirol 24:323–331. 10.1007/s13365-018-0615-7 29508305 10.1007/s13365-018-0615-7 PMC5992248

[42] Dossier C, Bonneric S, Baudouin V, Kwon T, Prim B, Cambier A, Couderc A, Moreau C, Deschenes G, Hogan J (2023) Obinutuzumab in frequently relapsing and steroid-dependent nephrotic syndrome in children. Clin J Am Soc Nephrol 18:1555–1562. 10.2215/CJN.0000000000000288 37678236 10.2215/CJN.0000000000000288 PMC10723910

[43] Brogan P, Yeung RSM, Cleary G, Rangaraj S, Kasapcopur O, Hersh AO, Li S, Paripovic D, Schikler K, Zeft A, Bracaglia C, Eleftheriou D, Pordeli P, Melega S, Jamois C, Gaudreault J, Michalska M, Brunetta P, Cooper JC, Lehane PB (2022) Phase IIa global study evaluating rituximab for the treatment of pediatric patients with granulomatosis with polyangiitis or microscopic polyangiitis. Arthritis Rheum 74:124–133. 10.1002/art.41901 10.1002/art.41901 PMC9299798 34164952

[44] Guigonis V, Dallocchio A, Baudouin V, Dehennault M, Hachon-Le Camus C, Afanetti M, Groothoff J, Llanas B, Niaudet P, Nivet H, Raynaud N, Taque S, Ronco P, Bouissou F (2008) Rituximab treatment for severe steroid- or cyclosporine-dependent nephrotic syndrome: a multicentric series of 22 cases. Pediatr Nephrol 23:1269–1279. 10.1007/s00467-008-0814-1 18465150 10.1007/s00467-008-0814-1

[45] Kamei K, Ito S, Nozu K, Fujinaga S, Nakayama M, Sako M, Saito M, Yoneko M, Iijima K (2009) Single dose of rituximab for refractory steroid-dependent nephrotic syndrome in children. Pediatr Nephrol 24:1321–1328. 10.1007/s00467-009-1191-0 19421786 10.1007/s00467-009-1191-0

[46] Ravani P, Magnasco A, Edefonti A, Murer L, Rossi R, Ghio L, Benetti E, Scozzola F, Pasini A, Dallera N, Sica F, Belingheri M, Scolari F, Ghiggeri GM (2011) Short-term effects of rituximab in children with steroid- and calcineurin-dependent nephrotic syndrome: a randomized controlled trial. Clin J Am Soc Nephrol 6:1308–1315. 10.2215/CJN.09421010 21566104 10.2215/CJN.09421010 PMC3109926

[47] Magnasco A, Ravani P, Edefonti A, Murer L, Ghio L, Belingheri M, Benetti E, Murtas C, Messina G, Massella L, Porcellini MG, Montagna M, Regazzi M, Scolari F, Ghiggeri GM (2012) Rituximab in children with resistant idiopathic nephrotic syndrome. Clin J Am Soc Nephrol 23:1117–1124. 10.1681/ASN.2011080775 10.1681/ASN.2011080775 PMC3358759 22581994

[48] Ravani P, Ponticelli A, Siciliano C, Fornoni A, Magnasco A, Sica F, Bodria M, Caridi G, Wei C, Belingheri M, Ghio L, Merscher-Gomez S, Edefonti A, Pasini A, Montini G, Murtas C, Wang X, Muruve D, Vaglio A, Martorana D, Pani A, Scolari F, Reiser J, Ghiggeri GM (2013) Rituximab is a safe and effective long-term treatment for children with steroid and calcineurin inhibitor-dependent idiopathic nephrotic syndrome. Kidney Int 84:1025–1033. 10.1038/ki.2013.211 23739238 10.1038/ki.2013.211 PMC3816123

[49] Sun L, Xu H, Shen Q, Cao Q, Rao J, Liu H-M, Fang X-Y, Zhou L-J (2014) Efficacy of rituximab therapy in children with refractory nephrotic syndrome: a prospective observational study in Shanghai. World J Pediatr 10:59–63. 10.1007/s12519-014-0453-5 24464665 10.1007/s12519-014-0453-5

[50] Ahn YH, Kim SH, Han KH, Choi HJ, Cho H, Lee JW, Shin JI, Cho MH, Lee JH, Park YS, Ha I-S, Cheong HI, Kim SY, Lee SJ, Kang HG (2018) Efficacy and safety of rituximab in childhood-onset, difficult-to-treat nephrotic syndrome: a multicenter open-label trial in Korea. Medicine 97:e13157. 10.1097/MD.0000000000013157 30431588 10.1097/MD.0000000000013157 PMC6257685

[51] Basu B, Sander A, Roy B, Preussler S, Barua S, Mahapatra TKS, Schaefer F (2018) Efficacy of Rituximab vs tacrolimus in pediatric corticosteroid-dependent nephrotic syndrome: a randomized clinical trial. JAMA Pediatr 172:757–764. 10.1001/jamapediatrics.2018.1323 29913001 10.1001/jamapediatrics.2018.1323 PMC6142920

[52] Takahashi T, Okamoto T, Sato Y, Yamazaki T, Hayashi A, Aoyagi H, Ueno M, Kobayashi N, Uetake K, Nakanishi M, Ariga T (2019) Periodically repeated rituximab administrations in children with refractory nephrotic syndrome: 2-year multicenter observational study. Pediatr Nephrol 34:87–96. 10.1007/s00467-018-4063-7 30141179 10.1007/s00467-018-4063-7

[53] Kari JA, Alhasan KA, Albanna AS, Safdar OY, Shalaby MA, Böckenhauer D, El-Desoky SM (2020) Rituximab versus cyclophosphamide as first steroid-sparing agent in childhood frequently relapsing and steroid-dependent nephrotic syndrome. Pediatr Nephrol 35:1445–1453. 10.1007/s00467-020-04570-y 32337638 10.1007/s00467-020-04570-y

[54] Ravani P, Lugani F, Pisani I, Bodria M, Piaggio G, Bartolomeo D, Prunotto M, Ghiggeri GM (2020) Rituximab for very low dose steroid-dependent nephrotic syndrome in children: a randomized controlled study. Pediatr Nephrol 35:1437–1444. 10.1007/s00467-020-04540-4 32232637 10.1007/s00467-020-04540-4

[55] Ravani P, Pisani I, Bodria M, Caridi G, Degl’Innocenti ML, Ghiggeri GM (2020) Low-dose ofatumumab for multidrug-resistant nephrotic syndrome in children: a randomized placebo-controlled trial. Pediatr Nephrol 35:997–1003. 10.1007/s00467-020-04481-y 31993781 10.1007/s00467-020-04481-y

[56] Ravani P, Colucci M, Bruschi M, Vivarelli M, Cioni M, DiDonato A, Cravedi P, Lugani F, Antonini F, Prunotto M, Emma F, Angeletti A, Ghiggeri GM (2021) Human or chimeric monoclonal anti-CD20 antibodies for children with nephrotic syndrome: a superiority randomized trial. J Am Soc Nephrol 32:2652–2663. 10.1681/ASN.2021040561 34544820 10.1681/ASN.2021040561 PMC8722811

[57] Ravani P, Lugani F, Drovandi S, Caridi G, Angeletti A, Ghiggeri GM (2021) Rituximab vs low-dose mycophenolate mofetil in recurrence of steroid-dependent nephrotic syndrome in children and young adults: a randomized clinical trial. JAMA Pediatr 175:631–632. 10.1001/jamapediatrics.2020.6150 33616641 10.1001/jamapediatrics.2020.6150 PMC7900932

[58] Al Salloum AA, Al Herbish AJ, Al Hissi MA, Abdalla MS, Salim SB, Farhat AH, Shagal RA, Othman A, Alshaiban A, Temsah M-HA, Al-Eyadhy AA, Alhasan KA (2022) The outcome of rituximab in treating steroid dependent nephrotic syndrome.: histopathology and immunosuppressive drugs as predicting factors. Saudi Med J 43:760–764. 10.15537/smj.2022.43.7.20210727 35830996 10.15537/smj.2022.43.7.20210727 PMC9749693

[59] Mathew G, Sinha A, Ahmed A, Grewal N, Khandelwal P, Hari P, Bagga A (2022) Efficacy of rituximab versus tacrolimus in difficult-to-treat steroid-sensitive nephrotic syndrome: an open-label pilot randomized controlled trial. Pediatr Nephrol 37:3117–3126. 10.1007/s00467-022-05475-8 35286456 10.1007/s00467-022-05475-8 PMC8919684

[60] Wang L, Zhu J, Xia M, Hua R, Deng F (2022) Comparison of rituximab, cyclophosphamide, and tacrolimus as first steroid-sparing agents for complicated relapsing/steroid-dependent nephrotic syndrome in children: an evaluation of the health-related quality of life. Arch Med Sci 18:275–278. 10.5114/aoms/145587 35154548 10.5114/aoms/145587 PMC8826811

[61] Basu B, Erdmann S, Sander A, Mahapatra TKS, Meis J, Schaefer F (2023) Long-term efficacy and safety of rituximab versus tacrolimus in children with steroid dependent nephrotic syndrome. Kidney Int Rep 8:1575–1584. 10.1016/j.ekir.2023.05.022 37547526 10.1016/j.ekir.2023.05.022 PMC10403658

[62] Zhu Y, Wu L, Wang Y, Zhu Y-F, Peng Y, Fang S-H, Zhang L-D, Deng F (2023) Efficacy and safety of low-dose rituximab in treatment of pediatric nephrotic syndrome: a prospective randomized controlled trial. Zhongguo dang dai er ke za zhi [Chinese J Contemp Pediatrics] 25:606–611. 10.7499/j.issn.1008-8830.2301026 37382130 10.7499/j.issn.1008-8830.2301026 PMC10321428

[63] Cravedi P, Bigatti C, Kajana X, Verrina EE, Caridi G, Bruschi M, Ghiggeri GM, Angeletti A (2024) Efficacy and safety of subcutaneous rituximab in idiopathic nephrotic syndrome. Kidney Int Rep 9:3332–3334. 10.1016/j.ekir.2024.08.021 39534199 10.1016/j.ekir.2024.08.021 PMC11551118

[64] Liu J, Deng F, Wang X, Liu C, Sun S, Zhang R, Zhang A, Jiang X, Yan W, Dou Y, Zhang Y, Xie L, Qian B, Shen Q, Xu H (2024) Early rituximab as an add-on therapy in children with the initial episode of nephrotic syndrome. Kidney Int Rep 9:1220–1227. 10.1016/j.ekir.2024.02.1395 38707815 10.1016/j.ekir.2024.02.1395 PMC11069012

[65] Nozu K, Sako M, Tanaka S, Kano Y, Ohwada Y, Morohashi T, Hamada R, Ohtsuka Y, Oka M, Kamei K, Inaba A, Ito S, Sakai T, Kaito H, Shima Y, Ishikura K, Nakamura H, Nakanishi K, Horinouchi T, Konishi A, Omori T, Iijima K (2024) Rituximab in combination with cyclosporine and steroid pulse therapy for childhood-onset multidrug-resistant nephrotic syndrome: a multicenter single-arm clinical trial (JSKDC11 trial). Clin Exp Nephrol 28:337–348. 10.1007/s10157-023-02431-0 38010466 10.1007/s10157-023-02431-0 PMC10955017

[66] Sheng A-Q, Liu F, Li Q-Y, Dou Y-L, Zhang X-J, Zhao J-L, Huang L-F, He S-Y, Lu Z-H, Feng C-Y, Wang J-J, Shen H-J, Fu H-D, Yan W-L, Mao J-H (2025) The efficacy and safety of rituximab monotherapy in the new onset pediatric idiopathic nephrotic syndrome: a randomized controlled clinical trial. Ren Fail 47:2499902. 10.1080/0886022X.2025.2499902 40328661 10.1080/0886022X.2025.2499902 PMC12057790

[67] Sinha R, Pradhan S, Raut S, Banerjee S, Sarkar S, Akhtar S, Dasgupta D, Poddar S, Mandal M, Kamal VK, Chaudhury AR, Tse Y (2025) Single (375 mg/m(2)) vs. double dose of rituximab along with mycophenolate mofetil for children with steroid-dependent/frequently relapsing nephrotic syndrome: a multicentre open-label randomized controlled trial. Pediatr Nephrol 40:995–1004. 10.1007/s00467-024-06619-8 39729126 10.1007/s00467-024-06619-8

[68] Billing H, Rieger S, Ovens J, Süsal C, Melk A, Waldherr R, Opelz G, Tönshoff B (2008) Successful treatment of chronic antibody-mediated rejection with IVIG and rituximab in pediatric renal transplant recipients. Transplantation 86:1214–1221. 10.1097/TP.0b013e3181880b35 19005402 10.1097/TP.0b013e3181880b35

[69] Zarkhin V, Li L, Kambham N, Sigdel T, Salvatierra O, Sarwal MM (2008) A randomized, prospective trial of rituximab for acute rejection in pediatric renal transplantation. Am J Transplant 8:2607–2617. 10.1111/j.1600-6143.2008.02411.x 18808404 10.1111/j.1600-6143.2008.02411.x

[70] Billing H, Rieger S, Süsal C, Waldherr R, Opelz G, Wühl E, Tönshoff B (2012) IVIG and rituximab for treatment of chronic antibody-mediated rejection: a prospective study in paediatric renal transplantation with a 2-year follow-up. Transpl Int 25:1165–1173. 10.1111/j.1432-2277.2012.01544.x 22897111 10.1111/j.1432-2277.2012.01544.x

[71] Hadjinicolaou AV, Nisar MK, Parfrey H, Chilvers ER, Ostor AJ (2012) Non-infectious pulmonary toxicity of rituximab: a systematic review. Rheumatology (Oxford) 51:653–662. 10.1093/rheumatology/ker290 22157468 10.1093/rheumatology/ker290

[72] Wijsenbeek M, Suzuki A, Maher TM (2022) Interstitial lung diseases. Lancet 400:769–786. 10.1016/S0140-6736(22)01052-2 35964592 10.1016/S0140-6736(22)01052-2

[73] Pradhan SK, Nayak S (2024) Rituximab induced rare cystic lesion in lungs in a nephrotic child: a case report. Indian J Nephrol 34:528–532. 10.25259/IJN_576_20 39372608 10.25259/IJN_576_20 PMC11450870

[74] Mathew G, George AS, Deepthi RV, Rose W, Verghese VP, Varghese R, Veeraraghavan B, Agarwal I (2023) Epidemiology and outcomes of pneumococcal sepsis in children with nephrotic syndrome in a developing country. Pediatr Nephrol 38:131–137. 10.1007/s00467-022-05550-0 35425998 10.1007/s00467-022-05550-0 PMC9009986

[75] Schweitzer L, Miko BA, Pereira MR (2024) Infectious disease prophylaxis during and after immunosuppressive therapy. Kidney Int Rep 9:2337–2352. 10.1016/j.ekir.2024.04.043 39156157 10.1016/j.ekir.2024.04.043 PMC11328545

[76] Chan EYH, Wong SW, Lai FFY, Ho TW, Tong PC, Lai WM, Ma ALT, Yap DYH (2023) Long-term outcomes with rituximab as add-on therapy in severe childhood-onset lupus nephritis. Pediatr Nephrol 38:4001–4011. 10.1007/s00467-023-06025-6 10.1007/s00467-023-06025-6 37358717

[77] Chan EY, Lai FF, Ma AL, Chan TM (2024) Managing lupus nephritis in children and adolescents. Paediatr Drugs 26:145–161. 10.1007/s40272-023-00609-3 38117412 10.1007/s40272-023-00609-3

[78] Kamei K, Takahashi M, Fuyama M, Saida K, Machida H, Sato M, Ogura M, Ito S (2015) Rituximab-associated agranulocytosis in children with refractory idiopathic nephrotic syndrome: case series and review of literature. Nephrol Dial Transplant 30:91–96. 10.1093/ndt/gfu258 25085238 10.1093/ndt/gfu258

[79] Dunleavy K, Tay K, Wilson WH (2010) Rituximab-associated neutropenia. Semin Hematol 47:180–186. 10.1053/j.seminhematol.2010.01.009 20350665 10.1053/j.seminhematol.2010.01.009 PMC7304500

[80] Chan EY-h (2024) Rituximab-induced hypogammaglobulinemia in nephrotic syndrome: what is the true burden? Pediatr Nephrol 39:3129–3129. 10.1007/s00467-024-06371-z 38600220 10.1007/s00467-024-06371-z

[81] Colucci M, Carsetti R, Serafinelli J, Rocca S, Massella L, Gargiulo A, Lo Russo A, Capponi C, Cotugno N, Porzio O, Onetti Muda A, Palma P, Emma F, Vivarelli M (2019) Prolonged impairment of immunological memory after anti-CD20 treatment in pediatric idiopathic nephrotic syndrome. Front Immunol 10:1653. 10.3389/fimmu.2019.01653 31379849 10.3389/fimmu.2019.01653 PMC6646679

[82] Kemper MJ, Altrogge H, Ganschow R, Muller-Wiefel DE (2002) Serum levels of immunoglobulins and IgG subclasses in steroid sensitive nephrotic syndrome. Pediatr Nephrol 17:413–417. 10.1007/s00467-001-0817-7 12107805 10.1007/s00467-001-0817-7

[83] Settipane GA, Pudupakkam RK, McGowan JH (1978) Corticosteroid effect on immunoglobulins. J Allergy Clin Immunol 62:162–166. 10.1016/0091-6749(78)90101-x 681628 10.1016/0091-6749(78)90101-x

[84] Parmentier C, Delbet J-D, Decramer S, Boyer O, Hogan J, Ulinski T (2020) Immunoglobulin serum levels in rituximab-treated patients with steroid-dependent nephrotic syndrome. Pediatr Nephrol 35:455–462. 10.1007/s00467-019-04398-1 31705306 10.1007/s00467-019-04398-1

[85] Marco H, Smith RM, Jones RB, Guerry M-J, Catapano F, Burns S, Chaudhry AN, Smith KG, Jayne DR (2014) The effect of rituximab therapy on immunoglobulin levels in patients with multisystem autoimmune disease. BMC Musculoskelet Disord 15:178. 10.1186/1471-2474-15-178 24884562 10.1186/1471-2474-15-178 PMC4038057

[86] Inoki Y, Kamei K, Nishi K, Sato M, Ogura M, Ishiguro A (2022) Incidence and risk factors of rituximab-associated hypogammaglobulinemia in patients with complicated nephrotic syndrome. Pediatr Nephrol 37:1057–1066. 10.1007/s00467-021-05304-4 34606002 10.1007/s00467-021-05304-4

[87] Kridin K, Ahmed AR (2020) Post-rituximab immunoglobulin M (IgM) hypogammaglobulinemia. Autoimmun Rev 19:102466. 10.1016/j.autrev.2020.102466 31917267 10.1016/j.autrev.2020.102466

[88] Shenoy M, Lennon R, Plant N, Wallace D, Kaur A (2020) Pre-emptive rituximab and plasma exchange does not prevent disease recurrence following living donor renal transplantation in high-risk idiopathic SRNS. Pediatr Nephrol 35:1081–1084. 10.1007/s00467-020-04500-y 32124030 10.1007/s00467-020-04500-y

[89] Chan EY, Webb H, Yu E, Ghiggeri GM, Kemper MJ, Ma AL, Yamamura T, Sinha A, Bagga A, Hogan J, Dossier C, Vivarelli M, Liu ID, Kamei K, Ishikura K, Saini P, Tullus K (2020) Both the rituximab dose and maintenance immunosuppression in steroid-dependent/frequently-relapsing nephrotic syndrome have important effects on outcomes. Kidney Int 97:393–401. 10.1016/j.kint.2019.09.033 31874801 10.1016/j.kint.2019.09.033

[90] Leung D, Chan EY-H, Mu X, Rosa Duque JS, Cheng SMS, Ho FT-W, Tong P-C, Lai W-M, Lee MHL, Chim S, Tam IYS, Tsang LCH, Kwan KKH, Chung Y, Wong HHW, Lee AMT, Li WY, Sze STK, Lam JHY, Lee DHL, Chan SM, Tu W, Peiris M, Ma AL-T, Lau YL (2023) Humoral and cellular immunogenicity of 3 doses of BNT162b2 in children with kidney diseases. Kidney Int Rep 8:2356–2367. 10.1016/j.ekir.2023.08.014 38025215 10.1016/j.ekir.2023.08.014 PMC10658278

[91] Ma AL, Leung D, Chan EY, Chim S, Cheng S, Ho FT, Lai WM, Tong PC, Lee MH, Wong WH, Chan SM, Rosa Duque J, Peiris JSM, Lau YL (2022) Antibody responses to 2 doses of mRNA COVID-19 vaccine in pediatric patients with kidney diseases. Kidney Int 101:1069–1072. 10.1016/j.kint.2022.01.035 35231464 10.1016/j.kint.2022.01.035 PMC8881810

[92] Iijima K, Sako M, Oba M, Tanaka S, Hamada R, Sakai T, Ohwada Y, Ninchoji T, Yamamura T, Machida H, Shima Y, Tanaka R, Kaito H, Araki Y, Morohashi T, Kumagai N, Gotoh Y, Ikezumi Y, Kubota T, Kamei K, Fujita N, Ohtsuka Y, Okamoto T, Yamada T, Tanaka E, Shimizu M, Horinochi T, Konishi A, Omori T, Nakanishi K, Ishikura K, Ito S, Nakamura H, Nozu K, Japanese Study Group of Kidney Disease in Children (2022) Mycophenolate mofetil after rituximab for childhood-onset complicated frequently-relapsing or steroid-dependent nephrotic syndrome. J Am Soc Nephrol 33:401–419. 10.1681/ASN.2021050643 34880074 10.1681/ASN.2021050643 PMC8819987

[93] Kado R, Sanders G, McCune WJ (2016) Suppression of normal immune responses after treatment with rituximab. Curr Opin Rheumatol 28:251–258. 10.1097/BOR.0000000000000272 27027812 10.1097/BOR.0000000000000272

[94] Venhoff N, Effelsberg NM, Salzer U, Warnatz K, Peter HH, Lebrecht D, Schlesier M, Voll RE, Thiel J (2012) Impact of rituximab on immunoglobulin concentrations and B cell numbers after cyclophosphamide treatment in patients with ANCA-associated vasculitides. PLoS One 7:e37626. 10.1371/journal.pone.0037626 22629432 10.1371/journal.pone.0037626 PMC3357389

[95] Hogan J, Godron A, Baudouin V, Kwon T, Harambat J, Deschenes G, Niel O (2018) Combination therapy of rituximab and mycophenolate mofetil in childhood lupus nephritis. Pediatr Nephrol 33:111–116. 10.1007/s00467-017-3767-4 28780657 10.1007/s00467-017-3767-4

[96] McGregor JG, Hogan SL, Kotzen ES, Poulton CJ, Hu Y, Negrete-Lopez R, Kidd JM, Katsanos SL, Bunch DO, Nachman PH, Falk RJ (2015) Rituximab as an immunosuppressant in antineutrophil cytoplasmic antibody-associated vasculitis. Nephrol Dial Transplant 30(Suppl 1):i123-131. 10.1093/ndt/gfv076 25805743 10.1093/ndt/gfv076 PMC4447867

[97] Kamar N, Milioto O, Puissant-Lubrano B, Esposito L, Pierre MC, Mohamed AO, Lavayssiere L, Cointault O, Ribes D, Cardeau I, Nogier MB, Durand D, Abbal M, Blancher A, Rostaing L (2010) Incidence and predictive factors for infectious disease after rituximab therapy in kidney-transplant patients. Am J Transplant 10:89–98. 10.1111/j.1600-6143.2009.02785.x 19656128 10.1111/j.1600-6143.2009.02785.x

[98] Gulleroglu K, Baskin E, Moray G, Ozdemir H, Arslan H, Haberal M (2016) Rituximab therapy and infection risk in pediatric renal transplant patients. Exp Clin Transplant 14:172–175. 10.6002/ect.2014.0156 26742572 10.6002/ect.2014.0156

[99] Furer V, Rondaan C, Heijstek MW, Agmon-Levin N, Assen Sv, Bijl M, Breedveld FC, D'Amelio R, Dougados M, Kapetanovic MC, Laar JMv, Thurah Ad, Landewé RB, Molto A, Müller-Ladner U, Schreiber K, Smolar L, Walker J, Warnatz K, Wulffraat NM, Elkayam O (2020) 2019 update of EULAR recommendations for vaccination in adult patients with autoimmune inflammatory rheumatic diseases. Ann Rheum Dis 79:39–52. 10.1136/annrheumdis-2019-215882 10.1136/annrheumdis-2019-215882 31413005

[100] Rubin LG, Levin MJ, Ljungman P, Davies EG, Avery R, Tomblyn M, Bousvaros A, Dhanireddy S, Sung L, Keyserling H, Kang I (2014) 2013 IDSA clinical practice guideline for vaccination of the immunocompromised host. Clin Infect Dis 58:e44–e100. 10.1093/cid/cit684 24311479 10.1093/cid/cit684

[101] Araujo-Neto JM, Guimaraes GS, Fernandes FF, Soares MA (2022) Hepatitis B surface antibody (Anti-HBs) kinetics during rituximab chemotherapy and performance of hepatitis B vaccine before immunosuppression: two prospective studies. Viruses 14:1780. 10.3390/v14081780 36016402 10.3390/v14081780 PMC9415137

[102] Carvajal R, Guananga-Alvarez D, Tur C, Esperalba J, Rodriguez-Barranco M, Rando-Segura A, Borras-Bermejo B, Cobo-Calvo A, Carbonell-Mirabent P, Zules-Ona R, Rodrigo-Pendas JA, Martinez-Gomez X, Montalban X, Tintore M, Otero-Romero S (2025) Effect of the number of vaccine doses before starting anti-CD20 therapy on seroprotection rates against hepatitis B virus in people with MS. Neurology 104:e210281. 10.1212/WNL.0000000000210281 39819099 10.1212/WNL.0000000000210281 PMC11737845

[103] Eisenberg RA, Jawad AF, Boyer J, Maurer K, McDonald K, Prak ETL, Sullivan KE (2013) Rituximab-treated patients have a poor response to influenza vaccination. J Clin Immunol 33:388–396. 10.1007/s10875-012-9813-x 23064976 10.1007/s10875-012-9813-x PMC3565069

[104] Mrak D, Tobudic S, Koblischke M, Graninger M, Radner H, Sieghart D, Hofer P, Perkmann T, Haslacher H, Thalhammer R, Winkler S, Blüml S, Stiasny K, Aberle JH, Smolen JS, Heinz LX, Aletaha D, Bonelli M (2021) SARS-CoV-2 vaccination in rituximab-treated patients: B cells promote humoral immune responses in the presence of T-cell-mediated immunity. Ann Rheum Dis 80:1345–1350. 10.1136/annrheumdis-2021-220781 34285048 10.1136/annrheumdis-2021-220781

[105] Stefanski A-L, Rincon-Arevalo H, Schrezenmeier E, Karberg K, Szelinski F, Ritter J, Jahrsdörfer B, Schrezenmeier H, Ludwig C, Sattler A, Kotsch K, Chen Y, Claußnitzer A, Haibel H, Proft F, Guerra G, Durek P, Heinrich F, Ferreira-Gomes M, Burmester GR, Radbruch A, Mashreghi M-F, Lino AC, Dörner T (2022) B cell numbers predict humoral and cellular response upon SARS-CoV-2 vaccination among patients treated with rituximab. Arthritis Rheum 74:934–947. 10.1002/art.42060 10.1002/art.42060 PMC9011692 34962360

[106] Lu L, Chan CY, Lim YY, Than M, Teo S, Lau PYW, Ng KH, Yap HK (2023) SARS-CoV-2 humoral immunity persists following rituximab therapy. Vaccines 11:1864. 10.3390/vaccines11121864 38140267 10.3390/vaccines11121864 PMC10748262

[107] Blanchard-Rohner G (2021) Vaccination in children with autoimmune disorders and treated with various immunosuppressive regimens: a comprehensive review and practical guide. Front Immunol 12:711637. 10.3389/fimmu.2021.711637 34408752 10.3389/fimmu.2021.711637 PMC8365419

[108] Caldera F, Mercer M, Samson SI, Pitt JM, Hayney MS (2021) Influenza vaccination in immunocompromised populations: strategies to improve immunogenicity. Vaccine 39(Suppl 1):A15–A23. 10.1016/j.vaccine.2020.11.037 33422377 10.1016/j.vaccine.2020.11.037

[109] Smith RM, Cooper DJ, Doffinger R, Stacey H, Al-Mohammad A, Goodfellow I, Baker S, Lear S, Hosmilo M, Pritchard N, Torpey N, Jayne D, Yiu V, Chalisey A, Lee J, Vilnar E, Cheung CK, Jones RB (2022) SARS-COV-2 vaccine responses in renal patient populations. BMC Nephrol 23:199. 10.1186/s12882-022-02792-w 35641961 10.1186/s12882-022-02792-w PMC9153874

[110] Vijenthira A, Gong I, Betschel SD, Cheung M, Hicks LK (2021) Vaccine response following anti-CD20 therapy: a systematic review and meta-analysis of 905 patients. Blood Adv 5:2624–2643. 10.1182/bloodadvances.2021004629 34152403 10.1182/bloodadvances.2021004629 PMC8216656

[111] Papp KA, Haraoui B, Kumar D, Marshall JK, Bissonnette R, Bitton A, Bressler B, Gooderham M, Ho V, Jamal S, Pope JE, Steinhart AH, Vinh DC, Wade J (2019) Vaccination guidelines for patients with immune-mediated disorders on immunosuppressive therapies. J Cutan Med Surg 23:50–74. 10.1177/1203475418811335 30463418 10.1177/1203475418811335 PMC6330697

[112] Kamei K (2023) Live attenuated vaccines in patients receiving immunosuppressive agents. Pediatr Nephrol 38:3889–3900. 10.1007/s00467-023-05969-z 37076756 10.1007/s00467-023-05969-z PMC10115603

[113] Government of Canada (2024) Hepatitis B vaccines: Canadian Immunization Guide. https://www.canada.ca/en/public-health/services/publications/healthy-living/canadian-immunization-guide-part-4-active-vaccines/page-7-hepatitis-b-vaccine.html. Accessed 13 August 2025

[114] Petrosky E, Bocchini JA, Hariri S, Chesson H, Curtis CR, Saraiya M, Unger ER, Markowitz LE, Centers for Disease Control and Prevention (CDC) (2015) Use of 9-valent human papillomavirus (HPV) vaccine: updated HPV vaccination recommendations of the advisory committee on immunization practices. MMWR Morb Mortal Wkly Rep 64:300–304 25811679 PMC4584883

[115] Panagiotakopoulos L, Moulia DL, Godfrey M, Link-Gelles R, Roper L, Havers FP, Taylor CA, Stokley S, Talbot HK, Schechter R, Brooks O, Daley MF, Fleming-Dutra KE, Wallace M (2024) Use of COVID-19 vaccines for persons aged ≥6 months: recommendations of the Advisory Committee on Immunization Practices - United States, 2024-2025. MMWR Morb Mortal Wkly Rep 73:819–824. 10.15585/mmwr.mm7337e2 39298394 10.15585/mmwr.mm7337e2 PMC11412444

[116] Alhasan KA, Raina R, Boyer O, Koh J, Bonilla-Felix M, Sethi SK, Amer YS, Coccia P, Temsah MH, Exantus J, Khan SA, Zhong X, Koch V, Duzova A, Vasudevan A, McCulloch M, Allen U, Filler G, Montini G, International Pediatric Nephrology Association (2025) IPNA clinical practice recommendations on care of pediatric patients with pre-existing kidney disease during seasonal outbreak of COVID-19. Pediatr Nephrol 40:1795–1815. 10.1007/s00467-024-06565-5 39733391 10.1007/s00467-024-06565-5 PMC11946955

[117] Committee on Infectious Diseases, American Academy of Pediatrics (2024) Red Book: 2024–2027 report of the Committee on Infectious Diseases. American Academy of Pediatrics, Itasca, Illinois

[118] Roberts DM, Jones RB, Smith RM, Alberici F, Kumaratne DS, Burns S, Jayne DRW (2015) Immunoglobulin G replacement for the treatment of infective complications of rituximab-associated hypogammaglobulinemia in autoimmune disease: a case series. J Autoimmun 57:24–29. 10.1016/j.jaut.2014.11.004 25586449 10.1016/j.jaut.2014.11.004

[119] Cowan J, Na I-K, Gladiator A, Kamieniak M, Mustafa SS (2025) Patient-reported outcomes with subcutaneous immunoglobulin in secondary immunodeficiency. Front Immunol 16:1528414. 10.3389/fimmu.2025.1528414 40181959 10.3389/fimmu.2025.1528414 PMC11967276

[120] Dinleyici EC, Frey G, Kola E, Wippermann U, Bauhofer A, Staus A, Griffiths P, Azhharry M, Rohsiswatmo R (2023) Clinical efficacy of IgM-enriched immunoglobulin as adjunctive therapy in neonatal and pediatric sepsis: a systematic review and meta-analysis. Front Pediatr 11:1239014. 10.3389/fped.2023.1239014 37635792 10.3389/fped.2023.1239014 PMC10451087

[121] Chung SA, Langford CA, Maz M, Abril A, Gorelik M, Guyatt G, Archer AM, Conn DL, Full KA, Grayson PC, Ibarra MF, Imundo LF, Kim S, Merkel PA, Rhee RL, Seo P, Stone JH, Sule S, Sundel RP, Vitobaldi OI, Warner A, Byram K, Dua AB, Husainat N, James KE, Kalot MA, Lin YC, Springer JM, Turgunbaev M, Villa-Forte A, Turner AS, Mustafa RA (2021) 2021 American College of Rheumatology/Vasculitis Foundation guideline for the management of antineutrophil cytoplasmic antibody-associated vasculitis. Arthritis Rheumatol 73:1366–1383. 10.1002/art.41773 34235894 10.1002/art.41773 PMC12327957

[122] Otani IM, Lehman HK, Jongco AM, Tsao LR, Azar AE, Tarrant TK, Engel E, Walter JE, Truong TQ, Khan DA, Ballow M, Cunningham-Rundles C, Lu H, Kwan M, Barmettler S (2022) Practical guidance for the diagnosis and management of secondary hypogammaglobulinemia: a work group report of the AAAAI primary immunodeficiency and altered immune response committees. J Allergy Clin Immunol 149:1525–1560. 10.1016/j.jaci.2022.01.025 35176351 10.1016/j.jaci.2022.01.025

[123] Monahan R, Otani IM, Lehman HK, Mustafa SS (2025) A second look at secondary hypogammaglobulinemia. Ann Allergy Asthma Immunol 134:269–278. 10.1016/j.anai.2024.12.003 39674275 10.1016/j.anai.2024.12.003

[124] Cote J, Chaloult-Lavoie M, Poulin E, Hayes LA, Singbo MNU, Ouellet P, Pelland-Marcotte MC (2025) Incidence of adverse events related to intravenous immunoglobulin therapy in children. Transfusion 65:88–99. 10.1111/trf.18083 39654082 10.1111/trf.18083 PMC11747083

[125] Kobayashi RH, Rigas MT (2022) Immune globulin therapy and kidney disease: overview and screening, monitoring, and management recommendations. Am J Health Syst Pharm 79:1415–1423. 10.1093/ajhp/zxac139 35595720 10.1093/ajhp/zxac139 PMC9389421

[126] Fragoulis GE, Nikiphorou E, Dey M, Zhao SS, Courvoisier DS, Arnaud L, Atzeni F, Behrens GM, Bijlsma JW, Böhm P, Constantinou CA, Garcia-Diaz S, Kapetanovic MC, Lauper K, Luís M, Morel J, Nagy G, Polverino E, van Rompay J, Sebastiani M, Strangfeld A, de Thurah A, Galloway J, Hyrich KL (2023) 2022 EULAR recommendations for screening and prophylaxis of chronic and opportunistic infections in adults with autoimmune inflammatory rheumatic diseases. Ann Rheum Dis 82:742–753. 10.1136/ard-2022-223335 36328476 10.1136/ard-2022-223335

[127] Kotton CN, Kumar D, Manuel O, Chou S, Hayden RT, Danziger-Isakov L, Asberg A, Tedesco-Silva H, Humar A, Transplantation Society International CMV Consensus Group (2025) The fourth international consensus guidelines on the management of cytomegalovirus in solid organ transplantation. Transplantation 109:1066–1110. 10.1097/TP.0000000000005374 40200403 10.1097/TP.0000000000005374 PMC12180710

[128] Chung MC, Chen CH, Chang SS, Lee CY, Tian YC, Wu MY, Wang HH, Yu CC, Chen TW, Kao CC, Hsu CY, Chiang YJ, Wu MJ, Chen YT, Wu MS (2025) Prevention and management of cytomegalovirus infection and disease in kidney transplant: a consensus statement of the Transplantation Society of Taiwan. J Formos Med Assoc 124:104–111. 10.1016/j.jfma.2024.05.009 38777672 10.1016/j.jfma.2024.05.009

[129] Stinco M, Rubino C, Trapani S, Indolfi G (2021) Treatment of hepatitis B virus infection in children and adolescents. World J Gastroenterol 27:6053–6063. 10.3748/wjg.v27.i36.6053 34629819 10.3748/wjg.v27.i36.6053 PMC8476329

[130] Committee on Infectious Diseases (2025) Recommendations for prevention and control of influenza in children, 2025–2026: technical report. Pediatrics 156:e2025073622. 10.1542/peds.2025-073622 40717224 10.1542/peds.2025-073622

[131] Clemente Garulo D, Nunez-Cuadros E, Camacho Lovillo M, Calzada-Hernandez J, Guillen Martin S, Fernandez Silveira L, Lirola Cruz MJ, Tagarro A, Alcobendas Rueda RM, Lopez Lopez A, Satrustegi Aritziturri M, Calvo C (2023) Position statement on infection screening, prophylaxis, and vaccination in pediatric patients with rheumatic diseases and immunosuppressive therapies, part 2: infection prophylaxis. Eur J Pediatr 182:4271–4284. 10.1007/s00431-023-05080-3 37439850 10.1007/s00431-023-05080-3 PMC10570166

[132] Sterling TR, Njie G, Zenner D, Cohn DL, Reves R, Ahmed A, Menzies D, Horsburgh CR, Crane CM, Burgos M, LoBue P, Winston CA, Belknap R (2020) Guidelines for the treatment of latent tuberculosis infection: recommendations from the National Tuberculosis Controllers Association and CDC, 2020. MMWR Recomm Rep 69:1–11. 10.15585/mmwr.rr6901a1 32053584 10.15585/mmwr.rr6901a1 PMC7041302

[133] Lachiewicz AM, Srinivas ML (2019) Varicella-zoster virus post-exposure management and prophylaxis: a review. Prev Med Rep 16:101016. 10.1016/j.pmedr.2019.101016 31890472 10.1016/j.pmedr.2019.101016 PMC6931226

[134] Park JW, Curtis JR, Choi SR, Kim MJ, Ha YJ, Kang EH, Lee YJ, Lee EB (2023) Risk–benefit analysis of primary prophylaxis against *Pneumocystis jirovecii* pneumonia in patients with rheumatic diseases receiving rituximab. Arthritis Rheum 75:2036–2044. 10.1002/art.42541 10.1002/art.42541 37096489

[135] Avino LJ, Naylor SM, Roecker AM (2016) *Pneumocystis jirovecii* pneumonia in the non-HIV-infected population. Ann Pharmacother 50:673–679. 10.1177/1060028016650107 27242349 10.1177/1060028016650107

[136] Hughes WT, Rivera GK, Schell MJ, Thornton D, Lott L (1987) Successful intermittent chemoprophylaxis for *Pneumocystis carinii* pneumonitis. N Engl J Med 316:1627–1632. 10.1056/NEJM198706253162604 3495732 10.1056/NEJM198706253162604

[137] Chen RY, Li DW, Wang JY, Zhuang SY, Wu HY, Wu JJ, Qu JW, Sun N, Zhong C, Zhu C, Zhang M, Yu YT, Yuan XD (2022) Prophylactic effect of low-dose trimethoprim-sulfamethoxazole for *Pneumocystis jirovecii* pneumonia in adult recipients of kidney transplantation: a real-world data study. Int J Infect Dis 125:209–215. 10.1016/j.ijid.2022.10.004 36243280 10.1016/j.ijid.2022.10.004

[138] Chen JK, Guerci J, Corbo H, Richmond M, Martinez M (2023) Low-dose TMP-SMX for *Pneumocystis jirovecii* pneumonia prophylaxis in pediatric solid organ transplant recipients. J Pediatr Pharmacol Ther 28:123–128. 10.5863/1551-6776-28.2.123 37139252 10.5863/1551-6776-28.2.123 PMC10150902

[139] Park JW, Curtis JR, Jun KI, Kim TM, Heo DS, Ha J, Suh K-S, Lee K-W, Lee H, Yang J, Kim MJ, Choi Y, Lee EB (2022) Primary prophylaxis for *Pneumocystis jirovecii* pneumonia in patients receiving rituximab. Chest 161:1201–1210. 10.1016/j.chest.2021.11.007 34788668 10.1016/j.chest.2021.11.007

[140] Kronbichler A, Kerschbaum J, Gopaluni S, Tieu J, Alberici F, Jones RB, Smith RM, Jayne DRW (2018) Trimethoprim-sulfamethoxazole prophylaxis prevents severe/life-threatening infections following rituximab in antineutrophil cytoplasm antibody-associated vasculitis. Ann Rheum Dis 77:1440–1447. 10.1136/annrheumdis-2017-212861 29950327 10.1136/annrheumdis-2017-212861 PMC6161662

[141] Pawłowska M, Flisiak R, Gil L, Horban A, Hus I, Jaroszewicz J, Lech-Marańda E, Styczyński J (2019) Prophylaxis of hepatitis B virus (HBV) infection reactivation - recommendations of the Working Group for prevention of HBV reactivation. Clin Exp Hepatol 5:195–202. 10.5114/ceh.2019.87631 31598555 10.5114/ceh.2019.87631 PMC6781818

[142] Indolfi G, Abdel-Hady M, Bansal S, Debray D, Smets F, Czubkowski P, van der Woerd W, Samyn M, Jahnel J, Gupte G, Zellos A, Mozer-Glassberg Y, Verkade HJ, Sokal E, Fischler B (2020) Management of hepatitis B virus infection and prevention of hepatitis B virus reactivation in children with acquired immunodeficiencies or undergoing immune suppressive, cytotoxic, or biological modifier therapies. J Pediatr Gastroenterol Nutr 70:527–538. 10.1097/MPG.0000000000002628 31977956 10.1097/MPG.0000000000002628

[143] Ali FS, Nguyen MH, Hernaez R, Huang DQ, Wilder J, Piscoya A, Simon TG, Falck-Ytter Y (2025) AGA clinical practice guideline on the prevention and treatment of hepatitis B virus reactivation in at-risk individuals. Gastroenterology 168:267–284. 10.1053/j.gastro.2024.11.008 39863345 10.1053/j.gastro.2024.11.008

[144] Terrault NA, Lok ASF, McMahon BJ, Chang K-M, Hwang JP, Jonas MM, Brown RS, Bzowej NH, Wong JB (2018) Update on prevention, diagnosis, and treatment of chronic hepatitis B: AASLD 2018 hepatitis B guidance. Hepatology (Baltimore, MD) 67:1560–1599. 10.1002/hep.29800 29405329 10.1002/hep.29800 PMC5975958

[145] Lin K-M, Lin J-C, Tseng W-Y, Cheng T-T (2013) Rituximab-induced hepatitis C virus reactivation in rheumatoid arthritis. J Microbiol Immunol Infect 46:65–67. 10.1016/j.jmii.2011.12.020 22627098 10.1016/j.jmii.2011.12.020

[146] Foca M, Demirhan S, Munoz FM, Valencia Deray KG, Bocchini CE, Sharma TS, Sherman G, Muller WJ, Heald-Sargent T, Danziger-Isakov L, Blum S, Boguniewicz J, Bacon S, Joseph T, Smith J, Ardura MI, Su Y, Maron GM, Ferrolino J, Herold BC (2024) Multicenter analysis of valganciclovir prophylaxis in pediatric solid organ transplant recipients. Open Forum Infect Dis 11:ofae353. 10.1093/ofid/ofae353 38979014 10.1093/ofid/ofae353 PMC11229698

[147] Allen UD, Preiksaitis JK, AST Infectious Diseases Community of Practice (2019) Post-transplant lymphoproliferative disorders, Epstein-Barr virus infection, and disease in solid organ transplantation: guidelines from the American Society of Transplantation Infectious Diseases Community of Practice. Clin Transplant 33:e13652. 10.1111/ctr.13652 31230381 10.1111/ctr.13652

[148] Alfano G, Giaroni F, Fontana F, Neri L, Mosconi G, Mussini C, Guaraldi G, Cappelli G (2020) Rituximab in people living with HIV affected by immune-mediated renal diseases: a case-series. Int J STD AIDS 31:1426–1431. 10.1177/0956462420946662 33104497 10.1177/0956462420946662

[149] Sadiq U, Shrestha U, Guzman N (2023) Prevention of Opportunistic Infections in HIV/AIDS. StatPearls Publishing, Treasure Island, Florida 30020717

[150] Rath E, Bonelli M, Duftner C, Gruber J, Mandl P, Moazedi-Furst F, Pieringer H, Puchner R, Flick H, Salzer HJF, Weiss G, Winkler S, Skvara H, Moschen A, Hofer H, Feurstein J, Sautner J (2022) National consensus statement by the Austrian Societies for Rheumatology, Pulmonology, Infectiology, Dermatology and Gastroenterology regarding the management of latent tuberculosis and the associated utilization of biologic and targeted synthetic disease modifying antirheumatic drugs (DMARDs). Wien Klin Wochenschr 134:751–765. 10.1007/s00508-022-02062-7 36036323 10.1007/s00508-022-02062-7 PMC9684247

[151] O’Young CKY, Ho KM, So H, Mok TYW, Leung CC, Chau CH, Chan CK (2021) Recommendations on management of latent tuberculosis infection in patients initiating anti-tumor necrosis factor biologics. J Clin Rheumatol Immunol 21:51–57. 10.1142/s2661341721500012

[152] Cruz AT, Ahmed A, Mandalakas AM, Starke JR (2013) Treatment of latent tuberculosis infection in children. J Pediatric Infect Dis 2:248–258. 10.1093/jpids/pit030 10.1093/jpids/pit030 26619479

