# Supplementary material for: Infection prophylaxis following anti-CD20 monoclonal antibodies in childhood kidney diseases
Source: Pediatr Nephrol. 2026 Feb 23;41(9):2799–823. doi: 10.1007/s00467-026-07180-2 (PMC13423921; doi:10.1007/s00467-026-07180-2)
Supplement: Supplementary file 1 — (DOCX 68.2 KB) [file 467_2026_7180_MOESM1_ESM.docx]

**Supplementary material**

Supplementary Table 1. KDIGO and IPNA guidelines for the indications of anti-CD20 in paediatric kidney diseases

| Condition | Indication for rituximab | Dose & Route & Timing | Co-therapy Guidance & Monitoring Recommendations | Source | Level of Evidence |
| --- | --- | --- | --- | --- | --- |
| **Nephrotic Syndrome** | | | | | |
| FRNS | Patients with glucocorticoids-related serious AEs; with FR despite prednisone + glucocorticoid-sparing oral agents. | 375 mg/m^2^ × 1-4 doses, i.v., when in remission with glucocorticoids | Co-glucocorticoids for ≥2 weeks; Oral cyclophosphamide and levamisole depending on patient-related issues.  Monitor: CD20, IgG before and after; screen: HBV, TB before. | KDIGO [1] | 1B |
| FRNS | Patients with poor control after 1 course with ≥1 steroid-sparing agent(s), especially when non-adherence. (B) Preferable in ≥7-9 years old. (C) | 375 mg/m^2^ × 1-4 doses, i.v., at weekly intervals, repeated courses can be given, preferably with remission patients. (C) | Taper off oral PDN and other steroid-sparing agents within 2-3 months.  Monitor quarterly: CBC, LFTs, CD19 counts and %, IgG (at baseline, quarterly in the 1st year, then yearly), total B cell counts at baseline and 7 days post rituximab; screen: HBV, HCV, HIV, EBV, TB. | INPA [2] | B |
| SDNS | All patients | 375 mg/m^2^ × 1-4 doses, i.v., when in remission with glucocorticoids | Co glucocorticoids for ≥2 weeks; MMF, rituximab, CNIs, and to a lesser extent, oral cyclophosphaminde are preferred, depending on patient-related issues  Monitor: IgG before and after; screen: HBV, TB. | KDIGO [1] | 1B |
| SDNS | Same to INPA for FRNS | / | / | INPA [2] | B |
| SRNS | Patients with CNI-resistant with no clinical trials available. | 375 mg/m^2^ × 1-2 doses, i.v., 2 infusions at day 1 and 8 when with nephrotic-range proteinuria | Monitor: IgG before and after; screen: HBV. | KDIGO [1] | ungraded |
| SRNS | Patients who fail to achieve at least partial remission with CNIs (ungraded), with no clinical trials available. (C) | 375 mg/m^2^ × 2 doses, i.v., 1-2 infusions within 2 weeks | Monitor: IgG after, first-AM proteinuria and B cell counts upon PR/CM; screen: HBV, JCV, TB. | INPA [3] | C |
| **ANCA-associated vasculitis** | | | | | |
| New onset | Glucocorticoids + rituximab /cyclophosphamide for new-onset. Rituximab preferred in children and adolescents, premenopausal women and men concerned about their fertility, frail older adults, when glucocorticoid-sparing especially important, relapsing diseases, and PR3-ANCA disease. | 375 mg/m^2^ × 4 doses, i.v., 1 infusion per week for 4 weeks | Glucocorticoid taper or avacopan | KDIGO [4] | 1B |
|  |  | 1g, i.v., 2 infusions at weeks 0 & 2 | Glucocorticoid taper or avacopan | KDIGO  [4] | 1B |
|  |  | 375 mg/m^2^ × 4 doses, i.v., 1 infusion per week for 4 weeks | Cyclophosphamide i.v. 15mg/kg at weeks 0 and 2 + glucocorticoid taper  Monitor: cyclophosphamide treatment toxicity, in particular leukopenia. | KDIGO  [4] | 1B |
|  |  | 1g × 2 doses, i.v., 1 infusion at weeks 0 and 2 | Cyclophosphamide i.v. 500mg every 2 weeks for 6 courses + glucocorticoid taper  Monitor: cyclophosphamide treatment toxicity, in particular leukopenia. | KDIGO  [4] | 1B |
| Maintenance | Maintenance with either rituximab, or azathioprine and low-dose glucocorticoids. Rituximab preferred in relapsing, PR3-ANCA disease, frail older adults, when glucocorticoid-sparing especially important, and azathioprine allergy. | 500mg × 5 doses, i.v., 2 infusions at complete remission, and 1 infusion at mo 6, 12, and 18 | / | KDIGO  [4] | 1C |
|  |  | 1000mg × 5 doses, i.v., 1 infusion at induction of remission, and at mo 4, 8, 12, and 16 | / | KDIGO  [4] | 1C |
| Relapse | For reinduce treatment when experiencing relapse. Same to the "new onset" | / | / | KDIGO  [4] | 1C |
| Refractory disease | Refractory disease with an increase in glucocorticoid, + rituximab if cyclophosphamide induction used previously. | / | Increase in glucocorticoids. Plasma exchange can be considered | KDIGO  [4] | 1C |
| **Anti-GBM disease** | | | | | |
| Refractory disease | Rituximab can be tried in refractory anti-GBM disease | / | / | KDIGO [5] | ungraded |
| **Lupus nephritis** | | | | | |
| Refractory disease | Patients with persistent disease activity or inadequate response to initial standard-of-care therapy | / | / | KDIGO [6] | ungraded |
| **Antibody mediated rejection** | | | | | |
| Acute rejection | Patients with antibody-mediated acute rejection | / | (corticosteroids), (plasma exchange), (intravenous immunoglobulin), (lymphocyte-depleting antibody) | KDIGO [7] | 2C |

For the IPNA guideline, the quality of evidence was graded as High (A), Moderate (B), Low (C), Very low (D); for the KDIGO guideline, the strength of recommendation is indicated as Level 1 (We recommend) or Level 2 (We suggest), and the certainty of the supporting evidence is shown as High (A), Moderate (B), Low (C), Very low (D). KDIGO: Kidney Disease: Improving Global Outcomes; IPNA, International Pediatric Nephrology Association; FRNS, frequently relapsing nephrotic syndrome; SDNS, steroid-dependent nephrotic syndrome; SRNS, steroid-resistant nephrotic syndrome; CNIs, calcineurin inhibitors; AAV, ANCA-associated vasculitis; AEs, adverse events; HBV, hepatitis B virus; TB, tuberculosis; HCV, hepatitis C virus; HIV, human immunodeciency virus; JCV, John Cunningham virus; CBC, complete blood count; LFTs, liver function test; PDN, prednisolone/prednisone; PR/CM, partial remission/complete remission.

Supplementary Table 2. Reports of adverse events related to infection after anti-CD20 use in kidney disease in adults from prospective studies (See Supplementary_table_2.xlsx)

Supplementary Table 3. Reported rates of neutropenia and agranulocytosis after anti-CD20 in children from prospective studies

| Study | Study Design | Disease | anti-CD20 | No. of Patients Analysed | No. of anti-CD20 courses | Regimen per Course mg/m^2^ | Rate of neutropenia | Rate of agranulocytosis |  |
| --- | --- | --- | --- | --- | --- | --- | --- | --- | --- |
|  |  |  |  |  |  |  |  |  |  |
| Guigonis *et al.,* 2008 [8] | Prospective | SDNS and SRNS | Rituximab | 22 | 40 | 375-1500 | 1/22 (4.5%) | NR |  |
| Ravani *et al.,* 2013 [9] | Prospective | NS | Rituximab | 46 | 104 | 375-750 | 3/46 (6.5%) | NR |  |
| Iijima *et al.,* 2014 [10] | DBRCT | FRNS and SDNS | Rituximab | 48 | 24 | 1500 | 4/24 (16.7%) | NR |  |
| Ravani *et al.,* 2015 [11] | RCT | SDNS | Rituximab | 30 | 15 | 375 | 0/15 (0%) | 0/15 (0%) |  |
| Basu *et al.,* 2018 [12] | RCT | CDNS | Rituximab | 120 | 60 | 750-1500 | 1/60 (1.7%) | NR |  |
| Takahashi *et al.,* 2019 [13] | Prospective | FRNS and SDNS | Rituximab | 22 | 22 | 1500 | 0/22 (0%) | 1/22 (4.5%) |  |
| Kari *et al.,* 2020 [14] | Prospective | SSNS | Rituximab | 46 | 19 | 750 | 1/19 (5.3%) | NR |  |
| Ravani *et al.,* 2021 [15] | RCT | SDNS | Ofatumumab | 140 | 140 | 375 | 4/140 (2.9%) | NR |  |
| Mathew *et al.,* 2022 [16] | RCT | SSNS | Rituximab | 41 | 21 | 750 | 1/21 (4.8%) | NR |  |
| Zhu *et al.,* 2023 [17] | RCT | FRNS and SDNS | Rituximab | 29 | 29 | 775-1500 | 0/29 (0%) | 0/29 (0%) |  |
| Cravedi *et al.,* 2024 SC [18] | Prospective | SDNS | Rituximab | 13 | 13 | 375 | 0/13 (0%) | 0/13 (0%) |  |
| Liu *et al.,* 2024 [19] | Prospective | SSNS | Rituximab | 76 | 43 | 375 | 18/43 (41.9%) | 1/43 (2.3%) |  |
| Sheng *et al.,* 2025 [20] | RCT | NS | Rituximab | 24 | 12 | 1500 | 3/12 (25%) | 1/12 (8.3%) |  |
| Sinha *et al.,* 2025 [21] | RCT | FRNS and SDNS | Rituximab | 91 | 91 | 375-750 | 4/91 (4.4%) | 0/91 (0%) |  |

RCT, randomised controlled trials; DBRCT, double blind, randomised controlled trial; NS, nephrotic syndrome; SDNS, steroid dependent nephrotic syndrome; SRNS, steroid resistant nephrotic syndrome; FRNS, frequent relapse nephrotic syndrome; CDNS, corticosteroid-dependent nephrotic syndrome; SC, rituximab subcutaneous injection; NR, not reported.

Supplementary Table 4. Reported rates of neutropenia and agranulocytosis after anti-CD20 in adults from prospective studies

| Study | Study Design | anti-CD20 | No. of Patients Analysed | No. of anti-CD20 courses | Regimen per Course mg/m^2^ | | Rate of neutropenia^#^ |
| --- | --- | --- | --- | --- | --- | --- | --- |
|  |  |  |  |  |  |  |  |
| **ANCA associated vasculitis** | | | | | |  |  |
| Jones *et al.,* 2010. [8] | RCT | Rituximab | 44 | 33 | 1500 | | 2/33 (6.1%) |
| Miloslavsky *et al.,* 2014. [15] | DBRCT | Rituximab | 26 | 26 | 1500 | | 0/26 (0%) |
| Nagafuchi *et al.,* 2015. [16] | Prospective | Rituximab | 7 | 9 | 1500 | | 1/7 (14.3%) |
| Charles *et al.,* 2018. [9] | RCT | Rituximab | 162 | 162 | 500-2500* | | 1/162 (0.6%) |
| Charles *et al.,* 2020. [10] | DBRCT | Rituximab | 97 | 50 | 2000* | | 1/50 (2%) |
| Nagasaka *et al.,* 2023. [17] | Prospective | Rituximab | 79 | 79 | 750-2000 | | 3/79 (3.8%) |
| **Lupus nephritis** | | | | | | | |
| Rovin *et al.,* 2012. [18] | DBRCT | Rituximab | 144 | 72 | 4000* | | 2/72 (2.8%) |
| Kotagiri *et al.,* 2016. [19] | Prospective | Rituximab | 14 | 14 | 375 | | 0/14 (0%) |
| Furie *et al.,* 2022. [20] | DBRCT | obinutuzumab | 125 | 63 | 4000* | | 3/63 (4.8%) |
| Furie *et al.,* 2025. [21] | RCT | obinutuzumab | 271 | 135 | 5000* | | 17/135 (12.6%) |
| **Antibody mediated rejection** | | | | | | | |
| Ahmadi *et al.,* 2017. [22] | Prospective study | Rituximab | 11 | 6 | 1500 | | 4/6 (66.7%) |
| Ahmadi *et al.,* 2019. [23] | Prospective study | Rituximab | 32 | 24 | 750-1500 | | 7/24 (29.2%) |

RCT, randomised controlled trials; DBRCT, double blind, randomised controlled trial; ^#^, no agranulocytosis was reported in the adult population; *, fixed dose in mg.

Supplementary Table 5. Reported rates of hypogammaglobulinemia after rituximab in adults from prospective studies

| Study | No. of Patients Analysed | No. of anti-CD20 courses | Regimen per Course mg/m^2^ | Outcome measures | | Rate of Hypogammaglobulinemia | |  |
| --- | --- | --- | --- | --- | --- | --- | --- | --- |
|  |  |  |  |  |  |  |  |  |
| **ANCA-associated vasculitis** | | | | | | |  |  |
| Jones. *et al.,* 2010. [8] | 44 | 33 | 1500 | patient based | Low IgG at 12 months | 1/33 (3.0%) | |  |
| Charles. *et al.,* 2020. [10] | 97 | 50 | 2000* | patient based | Low IgG at 28 months | 10/50 (20.0%) | |  |
| Smith. *et al.,* 2020. [11] | 188 | 188 | 1500 | patient based | Low IgG during rituximab | 51/188 (27.1%) | |  |
| Simth. *et al.,* 2023. [12] | 170 | 85 | 5000* | patient based | Low IgG during rituximab | 36/85 (42.4%) | |  |
| **Lupus nephritis** | | | | | | |  |  |
| Li. *et al.,* 2009. [13] | 19 | 19 | 1000* | patient based | Low IgG during rituximab | 0/19 (0%) | |  |
| Furie. *et al.,* 2022. [22] | 125 | 63 | 4000* | patient based | Low IgG at 104 week | 5/57 (9%) | |  |
| **Nephrotic syndrome** | | | | | | |  |  |
| Yimamuyushan. *et al.,* 2024. [14] | 60 | 60 | 1500 | patient based | Low IgG during rituximab | 0/60 (0%) | |  |

*, fixed dose in mg.

**List of References**

1. Floege J, Gibson KL, Vivarelli M, Liew A, Radhakrishnan J, Rovin BH (2025) KDIGO 2025 Clinical Practice Guideline for the Management of Nephrotic Syndrome in Children. Kidney Int 107:S241-S289. <https://doi.org/10.1016/j.kint.2024.11.007>

2. Trautmann A, Boyer O, Hodson E, Bagga A, Gipson DS, Samuel S, Wetzels J, Alhasan K, Banerjee S, Bhimma R, Bonilla-Felix M, Cano F, Christian M, Hahn D, Kang HG, Nakanishi K, Safouh H, Trachtman H, Xu H, Cook W, Vivarelli M, Haffner D, Association IPN (2023) IPNA clinical practice recommendations for the diagnosis and management of children with steroid-sensitive nephrotic syndrome. Pediatr Nephrol 38:877-919. <https://doi.org/10.1007/s00467-022-05739-3>

3. Trautmann A, Vivarelli M, Samuel S, Gipson D, Sinha A, Schaefer F, Hui NK, Boyer O, Saleem MA, Feltran L, Müller-Deile J, Becker JU, Cano F, Xu H, Lim YN, Smoyer W, Anochie I, Nakanishi K, Hodson E, Haffner D, Association IPN (2020) IPNA clinical practice recommendations for the diagnosis and management of children with steroid-resistant nephrotic syndrome. Pediatr Nephrol 35:1529-1561. <https://doi.org/10.1007/s00467-020-04519-1>

4. Kidney Disease: Improving Global Outcomes AVWG (2024) KDIGO 2024 Clinical Practice Guideline for the Management of Antineutrophil Cytoplasmic Antibody (ANCA)-Associated Vasculitis. Kidney Int 105:S71-S116. <https://doi.org/10.1016/j.kint.2023.10.008>

5. Kidney Disease: Improving Global Outcomes Glomerular Diseases Work G (2021) KDIGO 2021 Clinical Practice Guideline for the Management of Glomerular Diseases. Kidney Int 100:S1-S276. <https://doi.org/10.1016/j.kint.2021.05.021>

6. Rovin BH, Ayoub IM, Chan TM, Liu Z-H, Mejía-Vilet JM, Floege J (2024) KDIGO 2024 Clinical Practice Guideline for the management of LUPUS NEPHRITIS. Kidney Int 105:S1-S69. <https://doi.org/10.1016/j.kint.2023.09.002>

7. Kidney Disease: Improving Global Outcomes Transplant Work G (2009) KDIGO clinical practice guideline for the care of kidney transplant recipients. Am J Transplant 9 Suppl 3:S1-155. <https://doi.org/10.1111/j.1600-6143.2009.02834.x>

8. Guigonis V, Dallocchio A, Baudouin V, Dehennault M, Hachon-Le Camus C, Afanetti M, Groothoff J, Llanas B, Niaudet P, Nivet H, Raynaud N, Taque S, Ronco P, Bouissou F (2008) Rituximab treatment for severe steroid- or cyclosporine-dependent nephrotic syndrome: a multicentric series of 22 cases. Pediatr Nephrol 23:1269-1279. <https://doi.org/10.1007/s00467-008-0814-1>

9. Ravani P, Ponticelli A, Siciliano C, Fornoni A, Magnasco A, Sica F, Bodria M, Caridi G, Wei C, Belingheri M, Ghio L, Merscher-Gomez S, Edefonti A, Pasini A, Montini G, Murtas C, Wang X, Muruve D, Vaglio A, Martorana D, Pani A, Scolari F, Reiser J, Ghiggeri GM (2013) Rituximab is a safe and effective long-term treatment for children with steroid and calcineurin inhibitor-dependent idiopathic nephrotic syndrome. Kidney Int 84:1025-1033. <https://doi.org/10.1038/ki.2013.211>

10. Iijima K, Sako M, Nozu K, Mori R, Tuchida N, Kamei K, Miura K, Aya K, Nakanishi K, Ohtomo Y, Takahashi S, Tanaka R, Kaito H, Nakamura H, Ishikura K, Ito S, Ohashi Y, Group RfC-oRNSRS (2014) Rituximab for childhood-onset, complicated, frequently relapsing nephrotic syndrome or steroid-dependent nephrotic syndrome: a multicentre, double-blind, randomised, placebo-controlled trial. Lancet (London, England) 384:1273-1281. <https://doi.org/10.1016/S0140-6736(14)60541-9>

11. Ravani P, Rossi R, Bonanni A, Quinn RR, Sica F, Bodria M, Pasini A, Montini G, Edefonti A, Belingheri M, De Giovanni D, Barbano G, Degl'Innocenti L, Scolari F, Murer L, Reiser J, Fornoni A, Ghiggeri GM (2015) Rituximab in Children with Steroid-Dependent Nephrotic Syndrome: A Multicenter, Open-Label, Noninferiority, Randomized Controlled Trial. J Am Soc Nephrol 26:2259-2266. <https://doi.org/10.1681/ASN.2014080799>

12. Basu B, Sander A, Roy B, Preussler S, Barua S, Mahapatra TKS, Schaefer F (2018) Efficacy of Rituximab vs Tacrolimus in Pediatric Corticosteroid-Dependent Nephrotic Syndrome: A Randomized Clinical Trial. JAMA Pediatr 172:757-764. <https://doi.org/10.1001/jamapediatrics.2018.1323>

13. Takahashi T, Okamoto T, Sato Y, Yamazaki T, Hayashi A, Aoyagi H, Ueno M, Kobayashi N, Uetake K, Nakanishi M, Ariga T (2019) Periodically repeated rituximab administrations in children with refractory nephrotic syndrome: 2-year multicenter observational study. Pediatr Nephrol 34:87-96. <https://doi.org/10.1007/s00467-018-4063-7>

14. Kari JA, Alhasan KA, Albanna AS, Safdar OY, Shalaby MA, Böckenhauer D, El-Desoky SM (2020) Rituximab versus cyclophosphamide as first steroid-sparing agent in childhood frequently relapsing and steroid-dependent nephrotic syndrome. Pediatr Nephrol 35:1445-1453. <https://doi.org/10.1007/s00467-020-04570-y>

15. Ravani P, Colucci M, Bruschi M, Vivarelli M, Cioni M, DiDonato A, Cravedi P, Lugani F, Antonini F, Prunotto M, Emma F, Angeletti A, Ghiggeri GM (2021) Human or Chimeric Monoclonal Anti-CD20 Antibodies for Children with Nephrotic Syndrome: A Superiority Randomized Trial. J Am Soc Nephrol 32:2652-2663. <https://doi.org/10.1681/ASN.2021040561>

16. Mathew G, Sinha A, Ahmed A, Grewal N, Khandelwal P, Hari P, Bagga A (2022) Efficacy of rituximab versus tacrolimus in difficult-to-treat steroid-sensitive nephrotic syndrome: an open-label pilot randomized controlled trial. Pediatr Nephrol 37:3117-3126. <https://doi.org/10.1007/s00467-022-05475-8>

17. Zhu Y, Wu L, Wang Y, Zhu Y-F, Peng Y, Fang S-H, Zhang L-D, Deng F (2023) [Efficacy and safety of low-dose rituximab in treatment of pediatric nephrotic syndrome: a prospective randomized controlled trial]. Zhongguo dang dai er ke za zhi = Chinese journal of contemporary pediatrics 25:606-611. <https://doi.org/10.7499/j.issn.1008-8830.2301026>

18. Cravedi P, Bigatti C, Kajana X, Verrina EE, Caridi G, Bruschi M, Ghiggeri GM, Angeletti A (2024) Efficacy and Safety of Subcutaneous Rituximab in Idiopathic Nephrotic Syndrome. Kidney Int Rep 9:3332-3334. <https://doi.org/10.1016/j.ekir.2024.08.021>

19. Liu J, Deng F, Wang X, Liu C, Sun S, Zhang R, Zhang A, Jiang X, Yan W, Dou Y, Zhang Y, Xie L, Qian B, Shen Q, Xu H (2024) Early Rituximab as an Add-On Therapy in Children With the Initial Episode of Nephrotic Syndrome. Kidney Int Rep 9:1220-1227. <https://doi.org/10.1016/j.ekir.2024.02.1395>

20. Sheng A-Q, Liu F, Li Q-Y, Dou Y-L, Zhang X-J, Zhao J-L, Huang L-F, He S-Y, Lu Z-H, Feng C-Y, Wang J-J, Shen H-J, Fu H-D, Yan W-L, Mao J-H (2025) The efficacy and safety of rituximab monotherapy in the new onset pediatric idiopathic nephrotic syndrome: a randomized controlled clinical trial. Ren Fail 47:2499902. <https://doi.org/10.1080/0886022X.2025.2499902>

21. Sinha R, Pradhan S, Raut S, Banerjee S, Sarkar S, Akhtar S, Dasgupta D, Poddar S, Mandal M, Kamal VK, Chaudhury AR, Tse Y (2025) Single (375 mg/m(2)) vs. double dose of rituximab along with mycophenolate mofetil for children with steroid-dependent/frequently relapsing nephrotic syndrome: a multicentre open-label randomized controlled trial. Pediatr Nephrol 40:995-1004. <https://doi.org/10.1007/s00467-024-06619-8>

22. Furie RA, Aroca G, Cascino MD, Garg JP, Rovin BH, Alvarez A, Fragoso-Loyo H, Zuta-Santillan E, Schindler T, Brunetta P, Looney CM, Hassan I, Malvar A (2022) B-cell depletion with obinutuzumab for the treatment of proliferative lupus nephritis: a randomised, double-blind, placebo-controlled trial. Ann Rheum Dis 81:100-107. <https://doi.org/10.1136/annrheumdis-2021-220920>
