# Supplementary material for: Infection prophylaxis following anti-CD20 monoclonal antibodies in childhood kidney diseases
Source: Pediatr Nephrol. 2026 Feb 23;41(9):2799–823. doi: 10.1007/s00467-026-07180-2 (PMC13423921; doi:10.1007/s00467-026-07180-2)
Supplement: Supplementary file 3 — Graphical abstract (PPTX 213 KB) [file 467_2026_7180_MOESM3_ESM.pptx]

## Slide 1
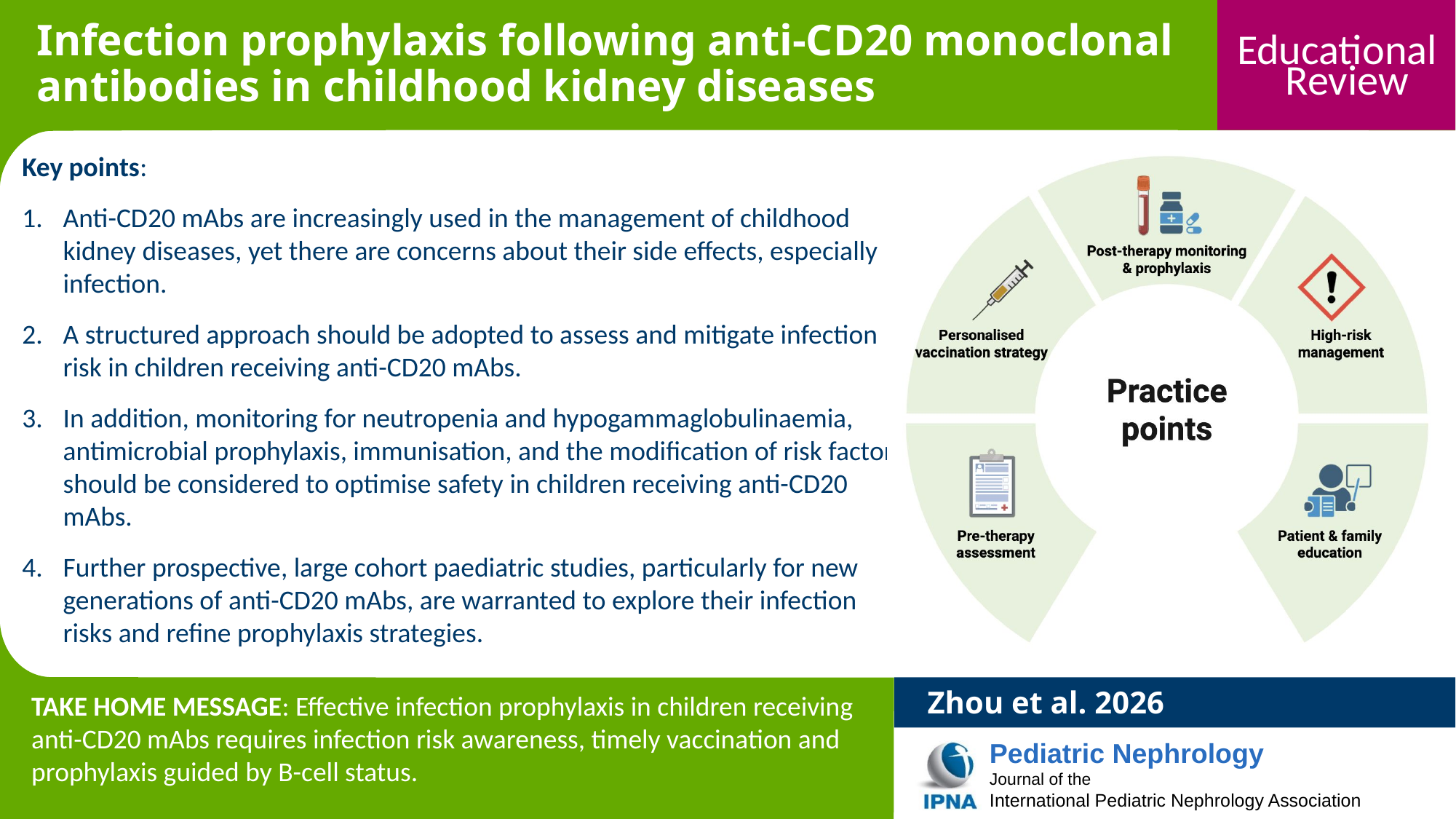

Infection prophylaxis following anti-CD20 monoclonal antibodies in childhood kidney diseases
Key points:
Anti-CD20 mAbs are increasingly used in the management of childhood kidney diseases, yet there are concerns about their side effects, especially infection.
A structured approach should be adopted to assess and mitigate infection risk in children receiving anti-CD20 mAbs.
In addition, monitoring for neutropenia and hypogammaglobulinaemia, antimicrobial prophylaxis, immunisation, and the modification of risk factors should be considered to optimise safety in children receiving anti-CD20 mAbs.
Further prospective, large cohort paediatric studies, particularly for new generations of anti-CD20 mAbs, are warranted to explore their infection risks and refine prophylaxis strategies.
Zhou et al. 2026
TAKE HOME MESSAGE: Effective infection prophylaxis in children receiving anti-CD20 mAbs requires infection risk awareness, timely vaccination and prophylaxis guided by B-cell status.
